# Gut colonization with an obesity-associated enteropathogenic microbe modulates the premetastatic niches to promote breast cancer lung and liver metastasis

**DOI:** 10.3389/fimmu.2023.1194931

**Published:** 2023-07-12

**Authors:** Sheetal Parida, Sumit Siddharth, Himavanth R. Gatla, Shaoguang Wu, Guannan Wang, Kathleen Gabrielson, Cynthia L. Sears, Brian H. Ladle, Dipali Sharma

**Affiliations:** ^1^ Department of Oncology, Sidney Kimmel Comprehensive Cancer Center, Baltimore, MD, United States; ^2^ Department of Oncology, Georgetown University, Baltimore, MD, United States; ^3^ Department of Medicine, Johns Hopkins University School of Medicine, Baltimore, MD, United States; ^4^ Johns Hopkins University School of Medicine, Molecular and Comparative Pathobiology, Baltimore, MD, United States; ^5^ Bloomberg-Kimmel Institute for Cancer Immunotherapy, Johns Hopkins University School of Medicine, Baltimore, MD, United States

**Keywords:** *B. fragilis*, ETBF, breast cancer, metastasis, immune microenvironment

## Abstract

**Introduction:**

Obesity, an independent risk factor for breast cancer growth and metastatic progression, is also closely intertwined with gut dysbiosis; and both obese state and dysbiosis promote each other. Enteric abundance of *Bacteroides fragilis* is strongly linked with obesity, and we recently discovered the presence of *B. fragilis* in malignant breast cancer. Given that enterotoxigenic *B. fragilis* or ETBF, which secretes *B. fragilis* toxin (BFT), has been identified as a procarcinogenic microbe in breast cancer, it is necessary to examine its impact on distant metastasis and underlying systemic and localized alterations promoting metastatic progression of breast cancer.

**Methods:**

We used syngeneic mammary intraductal (MIND) model harboring gut colonization with ETBF to query distant metastasis of breast cancer cells. Alterations in the immune network and cytokines/chemokines in the tumor microenvironment and distant metastatic sites were examined using flow cytometry, immunohistochemistry, and multiplex arrays.

**Results:**

ETBF infection initiates a systemic inflammation aiding in the establishment of the premetastatic niche formation in vital organs via increased proinflammatory and protumorigenic cytokines like IL17A, IL17E, IL27p28, IL17A/F, IL6, and IL10 in addition to creating a prometastatic immunosuppressive environment in the liver and lungs rich in myeloid cells, macrophages, and T regulatory cells. It induces remodeling of the tumor microenvironment via immune cell and stroma infiltration, increased vasculogenesis, and an EMT-like response, thereby encouraging early metastatic dissemination ready to colonize the conducive environment in liver and lungs of the breast tumor-bearing mice.

**Discussion:**

In this study, we show that enteric ETBF infection concomitantly induces systemic inflammation, reshapes the tumor immune microenvironment, and creates conducive metastatic niches to potentiate early dissemination and seeding of metastases to liver and lung tissues in agreement with the “seed and soil hypothesis.” Our results also support the ETBF-induced “parallel model” of metastasis that advocates for an early dissemination of tumor cells that form metastatic lesions independent of the primary tumor load.

## Graphical Abstract

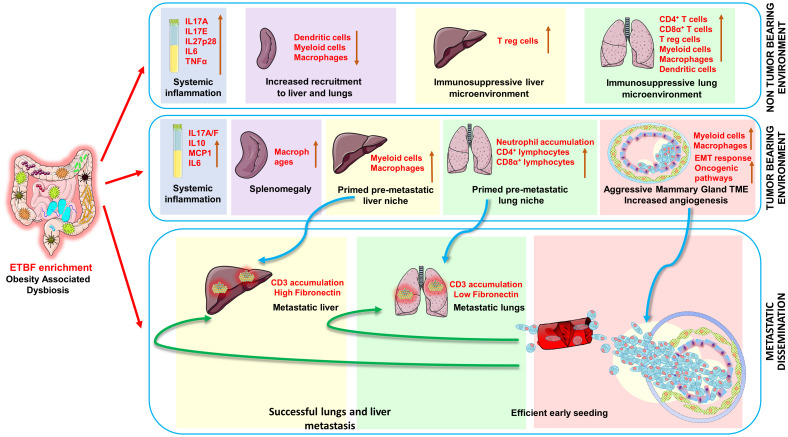

## Background

The impact of obesity on breast cancer can be appreciated by the fact that a 5-unit increase in body mass index (BMI) associates with a 12% increase in breast cancer risk, and approximately 13% of adults worldwide and 41.9% of adults in the United States are obese (BMI >30) (https://www.cdc.gov/obesity/data/adult.html). Obesity is also strongly interconnected with gut dysbiosis, a state characterized by low diversity and enrichment of pathogenic bacteria. Obesity and dysbiosis influence each other; while obese state is accentuated by certain microbes supporting increased metabolism and enhanced energy harvest via elevated production of short-chain fatty acids (SCFAs), absorption of monosaccharides, and microbial fermentation of indigestible polysaccharides, obese state, in turn, provides a conducive environment for pathogenic microbes (1, 2). Adiposity and altered body fat composition can significantly influence breast cancer outcomes through an upsurge of proinflammatory cytokines and immune modulation. Likewise, dysbiotic gut microbiota can also influence breast cancer via several means including small metabolite-induced oncogenic signaling, systemic inflammation, and altered hormone regulation (3, 4). Accordingly, several studies and clinical trials have implied the connection between obesity-induced dysbiosis and poor breast cancer outcomes (3, 5, 6), although the involvement of specific microbes is currently unclear.

It has been estimated that 13% of global cancer burden can be attributed to microbial infections (7). Of the 500–1,000 members of the microbiota and ~10^12^ known microbes on earth, only 10 have been characterized as Group I human carcinogens (8). While there is no dearth of data demonstrating the influence of microbiota in causation and therapeutic outcomes in colorectal and pancreatic cancers, the research in breast cancer is in its infancy. The insufficiency can be attributed to the fact that the breast has been considered a sterile organ until recently. Recent studies confirm the presence of a distinct breast microbiota that is clearly distinguishable between healthy and cancerous breast tissue (9–18). In fact, breast tumors are found to be the richest and most biodiverse among the nine tumor types examined, and the tumor-specific microbes reside within tumor as well as immune cells in a cell wall-deficient intracellular state (19). Translocation of oral microbes via the bloodstream into breast cancers has been confirmed (20). Also, distinct differences in gut microbiota have been observed between breast cancer patients and healthy controls, suggesting an association with breast cancer initiation and progression (21, 22). Interestingly, tumor-resident intratumoral bacteria may drive metastatic colonization of breast cancer by inducing cytoskeletal reorganization in circulating tumor cells, thereby enhancing resistance to mechanical stress in the circulation (23). Microbes are enzyme factories, and microbiota-derived metabolites, such as Lipopolysaccharide (LPS), have been shown to aggravate breast cancer (24). Moreover, bacterial quorum-sensing molecules have also been shown to enhance proliferation (25) and potentiate angiogenesis and invasion of breast cancer cells (26). Dysbiosis also influences metabolism and bioavailability of drugs, therefore regulating therapy response (27). A spontaneous mammary tumorigenesis model infected with *Helicobacter hepaticus* yields more aggressive multifocal mammary tumors with significantly higher accumulation of neutrophils around the periphery of the tumors and in the stroma, and interestingly, these protumor effects are inhibited by the elimination of neutrophils following antibody treatment (28). It seems that the effects of microbiota and its metabolites on breast cancers are mediated at least partially via the immune system, as the breast microbiota correlates with the immunological signatures of breast cancer and the extent and expression of tumor-infiltrating lymphocytes (29, 30). It is intriguing how microbes can influence the tumor immune microenvironment and influence tumor progression.

Drawing connections between obesity, microbiota, and breast cancer, increased abundance of Firmicutes and decreased levels of Bacteroidetes have been shown in the obese state (31) as well as in breast cancer versus normal breasts (9) and malignant breast cancer versus benign breast disease (1, 11). Overall, many microbial families and species enriched in the obese state are similar to those observed in breast cancer samples compared to normal breasts, suggesting a microbial link between obesity, dysbiosis, and breast cancer risk (1, 32). Dysbiosis leads to increased gut permeability resulting in elevated circulating LPS levels causing inflammation and insulin resistance. Systemic LPS stimulates Adipocyte fatty acid binding protein (A-FABP) secretion from adipose tissue via Toll like receptor 4 (TLR4) activation (33). Microbiota is also known to regulate the gut endocannabinoid system that can potentially stimulate an obese phenotype (34). *Bacteroides fragilis*, a common gut colonizer, has been associated with childhood and adolescent obesity (35, 36). Sun et al. (37) demonstrated that *B. fragilis* colonization in high-fat diet-fed mice led to more severe metabolic dysfunction and hyperglycemia mediated by increased bile acid glycoursodeoxycholic acid (GUDCA) via intestinal farnesoid X receptor (FXR) signaling, and the gold standard drug, metformin, mitigates metabolic dysfunction via the *B. fragilis*–GUDCA–intestinal FXR axis. Another recent study showed that artificial sweeteners associated with decreased gut microbial diversity; particularly, popular non-calorie sweetener, sucralose, led to increased intestinal *B. fragilis* abundance, leading to decreased abundance of occludin (an epithelial cell tight junction membrane protein). Interestingly, higher levels of ketone bodies, fatty acid oxidation, glucose intolerance, and an increase in proinflammatory cytokines are also observed. In combination with a high-fat diet, *B. fragilis* not only causes higher metabolic endotoxemia and weight gain, it also increases body fat, total SCFAs, serum Tumor necrosis factor-alpha (TNFα) concentration, and glucose intolerance (38). A decrease in obesity parameters including BMI, fat mass, blood glucose, blood lipid, and hepatic function is associated with a decrease in the abundance of *B. fragilis* in the gut (39). Using a stringent constrained linear regression analysis, Shen et al. (40) demonstrated the association of obesity with intestinal *B. fragilis* abundance in perimenopausal and postmenopausal women. *B. fragilis* accelerates obesity, in part, by suppressing acetic acid levels (40); acetates fuel the growth of health-associated microbes responsible for producing propionate and butyrate that maintain the integrity of the gut mucosa (41). These studies have established a strong link between the enteric abundance of *B. fragilis* and obesity.

Most interestingly, we recently discovered that the female breast is inhabited by *B. fragilis*, which is more abundant in cancerous compared to normal breast tissue. Our recent study has shown that enteric as well as mammary duct colonization with enterotoxigenic *B. fragilis* (ETBF), which secretes *B. fragilis* toxin (BFT), leads to mammary epithelial cell hyperplasia and higher breast cancer growth and stemness, and these oncogenic effects are mediated via β-catenin and Notch1 axes (42, 43). In the present study, we aim to decipher the impact of enteric ETBF infection on distant metastasis of breast cancer cells, specifically asking (i) whether ETBF causes increased metastasis; (ii) whether ETBF manipulates the premetastatic niche in the liver and lungs, supporting the establishment of metastatic lesions in these organs; and (iii) whether ETBF causes systemic inflammation and alters the tumor immune microenvironment. Our results demonstrate that gut colonization with ETBF accelerates early and efficient dissemination of breast cancer cells from the primary tumor, causes systemic inflammation, prepares premetastatic niches, modulates the tumor immune microenvironment, and aids in the development of metastatic lesions.

## Materials and methods

### Cell lines and bacterial strains

Luciferase-tagged mouse mammary cancer cell line 4T1-luc2, a generous gift from Dr. Saraswati Sukumar, Johns Hopkins University School of Medicine, was maintained in 5% CO_2_ atmosphere, 37°C temperature, and 95% humidity. The 4T1-luc2 cells were grown in 10% heat-inactivated Fetal bovine serum (FBS) supplemented in DMEM F/12 (Corning, USA). Cells were used for experiments within 10–20 passages from thawing. All cells were authenticated via short tandem repeat testing. *Mycoplasma* detection was routinely performed using the MycoAlert Detection Kit (Lonza, LT07-218). Cultures of enterotoxigenic *B. fragilis* (ETBF) strain 86-5443-2-2 (that secretes *B. fragilis* toxin, BFT) and its isogenic nontoxigenic mutant (incapable of secreting BFT owing to an in-frame deletion of the chromosomal *bft* gene) were maintained anaerobically at 37°C. Bacterial pellets were washed and resuspended with 1× Dulbecco PBS (1× PBS free of calcium chloride and magnesium chloride) for mouse inocula.

### Reagents

FPLC purification of culture supernatants of ETBF was conducted to purify BFT and stored at -80°C, as previously described (44). For immunohistochemistry, rabbit monoclonal anti-E-cadherin, anti-cMyc, anti-Notch1, anti-NICD, anti-Oct4, anti-Nanog, anti-KLF4, and anti-Ki67 were purchased from Cell Signaling Technology, Beverly, MA, USA. Anti-β-catenin, anti-CD31, anti-Jagged1 antibodies were purchased from Santa Cruz Biotechnology, Santa Cruz, CA, USA. IHC-specific rabbit monoclonal cMyc was purchased from Abcam, Cambridge, MA, USA. Mouse monoclonal β-actin was purchased from Sigma-Aldrich, St. Louis, MO, USA. Anti-IL6 and anti-TNFα antibodies for ELISA were obtained from Santa Cruz Biotechnology, Santa Cruz, CA, USA. For flow cytometry, anti-CD3, anti-CD4, anti-CD8, anti-CD45, anti-CD11c, anti-CD11b, anti-FOXP3, anti-PD-L1, anti-F4/80, anti-granzyme B, anti-IFNγ, and anti-TNFα antibodies were purchased from BD Biosciences, San Jose, CA. Horseradish peroxidase-conjugated goat anti-rabbit IgG, goat anti-mouse IgG, and donkey anti-goat IgG were purchased from Sigma-Aldrich, St. Louis, MO, USA. Chemiluminescent peroxidase substrate and 3-(4,5-dimethylthiazol-2-yl)-2,5 diphenyltetrazolium bromide (MTT) were also obtained from Sigma-Aldrich, St. Louis, MO, USA. Rhodamine phalloidin was procured from Invitrogen Corporation, CA, USA. FBS, 6-thioguanine, and D-luciferin were purchased from Sigma-Aldrich, St. Louis, MO, USA. Matrigel basement matrix was purchased from Corning Inc., NY, USA. MSD V-PLEX Proinflammatory Panel 1 ELISA kit for mouse cytokines was procured from Meso Scale Diagnostics, Rockville, MD, USA.

### ETBF gut colonization

All animal studies were in accordance with the guidelines of Johns Hopkins University ACUC. BALB/c mice were obtained from Charles River and maintained in-house. Mice were given antibiotic cocktail (clindamycin 0.1 g/L and streptomycin 5 g/L) in water bottles (Hospira and Amresco) for 7 days and discontinued. ETBF strain 86-5443-2-2 was cultured in BHI media at 37°C under anaerobic conditions for 24 h. Bacteria in their logarithmic growth phase were spun down, washed three times in sterile PBS, and resuspended in the same volume of sterile PBS. BALB/c mice were infected with ~10^8^ CFU of ETBF or 086Mut in PBS by oral gavage. For sham control, mice were gavaged with PBS (45).

### ETBF-4T1 mammary intraductal (MIND syngeneic model) implant and tumor monitoring

Twice parous BALB/c mice were procured from Charles River, maintained in-house, and were given antibiotic cocktail (clindamycin 0.1 g/L and streptomycin 5 g/L) in water bottles (Hospira and Amresco, Lake Forest, IL, USA) for 1 week and discontinued. Mice in *gut colonization of ETBF or 086Mut* experimental groups were then oral gavaged with ∼10^8^ CFU of ETBF or 086Mut strains of *B. fragilis* in PBS and allowed to colonize for 3 days. For the sham control group, mice were oral gavaged with PBS. Intraductal mammary tumors were established by injecting 5,000 4T1-Luc2 cells directly into the mammary ducts of mice on one side. IVIS spectrum at SKCCC animal resources was utilized to regularly monitor tumor progression by bioluminescence imaging. For bioluminescence imaging, mice were injected with 10 μl/g d-luciferin (15 mg/ml in PBS) intraperitoneally, and images were captured 8–10 min after injection. At the end of the experiment, *ex vivo* bioluminescence images of major organs, lungs, and liver were captured to investigate metastatic progression. Briefly, animals were given an intraperitoneal injection of D-luciferin and were euthanized after 10 min. Lungs and liver were excised, and images were captured using the IVIS system. Tumors were excised, weighed, and preserved for subsequent studies. Blood was collected by heart puncture in serum separator tubes and incubated at room temperature for 30 min, followed by centrifugation at 10,000 RPM for 15 min. Serum was transferred to microcentrifuge tubes and snap-frozen in liquid nitrogen.

### 4T1 metastasis assay

Lung and liver excised from 4T1-Luc2 tumor-bearing BALB/c mice were harvested in DMEM F12 medium. With curved scissors, lungs and liver were minced into pieces and transferred into 15-ml tubes containing 2.5 ml of the respective digestion cocktail; RPMI + 10 mg/ml collagenase A for liver and RPMI + 10 mg/ml of collagenase A + 10 mg/ml of hyaluronidase for lungs. The organs were then placed in shaking water bath at 37°C for 30 min to allow complete dissociation. After enzymatic digestion, volumes of the samples were made up to 10 ml with PBS and each sample was filtered through separate 70-μm nylon cell strainers to remove large chunks of undigested tissue. Samples were collected in 50-ml tubes, centrifuged for 5 min at 1,500 rpm, Room temperature (RT), in a benchtop centrifuge, and supernatant was discarded. Samples were washed twice by centrifugation in PBS. Pellets were resuspended in culture media containing 10 μl of 60 mM 6-thioguanine and plated onto 6-well culture plates containing 2-ml media. Plates were incubated in 37°C tissue culture incubator, 5% CO_2_ to allow growth of colonies for 3–7 days (46).

### Isolation of splenocytes and tumor-infiltrating lymphocytes for flowcytometry

For flow cytometric analysis of immune infiltrates, spleen, tumors, liver, and lungs were excised and immediately collected in harvest media (RPMI supplemented with 4% FBS and 1% antibiotic/antimycotic cocktail). *For isolating splenocytes*, single-cell suspensions were generated mashing the spleen through a 70-µm cell strainer. After washing with complete media (RPMI + 10% FBS + antibiotic/antimycotic cocktail), splenocytes were subjected to red cell lysis with ACK (ammonium-chloride-potassium) lysing buffer and again washed with complete media to generate single-cell suspensions for flow cytometry staining. *For isolation of tumor-infiltrating lymphocytes*, minced tumor tissues were processed in 5 ml of tumor digest media [RPMI supplemented 5% FBS, 0.7 mg/ml collagenase I (270 units/mg, Gibco, Grand Island, NY), 0.04 mg/ml of DNAse I (grade II, from bovine pancreas, Millipore Sigma)] and were incubated in rotating shaker for 30 min at 37°C followed by addition of 100 µl of 0.5 M EDTA to quench the digest process. The tumor digest was passed through 40-µm strainers, washed, and enriched for viable cells by Ficoll Paque plus density gradient separation. The single-cell suspension of tumors cells was washed with complete media. To stimulate the immune cells for cytokine detection, they were incubated for 4 h at 37°C in media with 50 ng/ml of phorbol-12-myristate-13-acetate (PMA), 0.5 µg/ml of ionomycin, and BD GolgiStop (BD Biosciences). Tumor infiltrating lymphocytes (TILs) and splenocytes were stained and analyzed by flow cytometry. The number of TILs per gram of tumor was calculated based on the weight of the tumor prior to digest, the number of viable cells obtained per tumor prior to antibody staining, and the percentage of viable cells of each cell type determined by flow cytometry analysis.

### ELISA

For IL6 and TNFα measurements, antigen capture ELISA was performed. Here, 10 μl of serum sample was used to coat the wells of a microtiter plate. The antigen was allowed to bind overnight at 4°C. The following morning, antigen blocking was done with 5% BSA and incubated with primary antibody for 4 h at room temperature. Antibody was flicked and washed with PBS four times. Horseradish peroxidase (HRP)-labeled secondary antibody was added to the wells and incubated for 2 h at room temperature. After four PBS washes, TMB liquid substrate was added and allowed to oxidize for about 20 min; the blue oxidized chromogen was read at 450 nm. Color intensity was proportional to antigen concentration. For multiplexed ELISA, MSD V-PLEX Mouse Cytokine 29-Plex Kit (Meso Scale Diagnostics, Rockville, MD, USA) was used as per manufacturer’s protocol. The 29 assays in the V-PLEX Mouse Cytokine 29-Plex are provided in three multiplex panels—Proinflammatory Panel 1 (mouse), Cytokine Panel 1 (mouse), and Th17 Panel 1 (mouse).

### Immunohistochemistry

Tumor, lung, liver, and spleen specimens excised from experimental animals were fixed in 10% formalin, paraffin-embedded, and sectioned. *For histology*, sections were deparaffinized, and H&E staining and Mason trichrome staining were performed. *For immunohistochemical analysis*, tissue sections were deparaffinized and rehydrated followed by antigen retrieval in citrate buffer. IHC for specific proteins was carried out on tumor tissue sections using Vectastain ABC detection system according to Vector Lab guidelines. Briefly, post antigen retrieval, tissue antigens were blocked with 10% normal goat serum for 30 min at room temperature. Sections were then incubated overnight with 100 times diluted primary antibodies (anti–Ki-67, anti–CD31, anti–β-catenin, anti-E-cadherin, anti-N-cadherin, and cMyc) in 10% normal goat serum at 4°C. After one PBS wash, sections were incubated with secondary antibody for 1 h at room temperature followed by 30 min of incubation with ABC reagent. After one PBS wash, DAB chromogen was added and incubated at room temperature from 2 to 7 min depending on signal intensity. Slides were counterstained with hematoxylin, dehydrated, and mounted. Images were captured using Nikon Eclipse Si microscope at 20× magnification. Images were annotated and analyzed using Leica-ImageScope software (42, 47) and the Aperio Toolbox (https://tmalab.jhmi.edu/aptk.html).

### Statistical analysis

All experiments were performed thrice in triplicate. Measurements of micrographs were done using Leica ImageScope software. Flow cytometry analysis was done using FlowJo software. IHC images were analyzed using Aperio ImageScope software. Quantification of Western blots was done using GelQuant.NET software. Statistical analysis was done using GraphPad Prism 9. Results were considered to be statistically significant if p < 0.05. Results were expressed as mean ± SEM between triplicate experiments performed thrice. For comparison between multiple groups, statistical significance was determined by one-way ANOVA and Bonferroni analysis. Comparisons between two groups were done using Student’s t-test.

## Results

### Gut colonization with ETBF stimulates early metastatic dissemination from mammary glands

**Figure 1:**
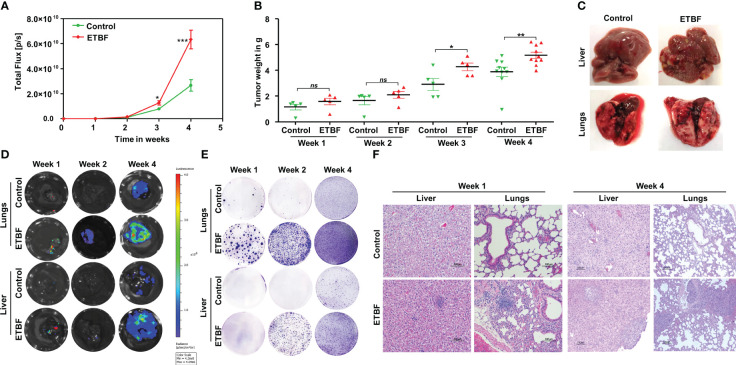
Gut colonization with ETBF enhances breast cancer growth and distant organ metastasis in the syngeneic mouse model. 4T1 intraductal breast tumor implant in BALB/c mice with ETBF gut colonization or sham control. **(A)** Tumor progression curves of 4T1 cells implanted intraductally in BALB/c mice harboring enteric ETBF gut colonization or sham control, represented by bioluminescence measured as total flux (n = 5). **(B)** Plot shows weights of 4T1 tumors from each experimental group for 4 weeks post-tumor cell implantation. **(C)** Representative images showing metastatic lesions in the liver and lungs in 4T1 intraductal tumor-bearing mice without or with enteric ETBF infection at 4 weeks. **(D)** *Ex situ* bioluminescence imaging showing early to late metastatic dissemination to the lungs and liver in sham control and ETBF-infected mice. **(E)** *Ex vivo* metastatic colony formation assay from resected organs at different points of tumor progression. **(F)** Representative H&E images showing the histology of the lungs and liver of sham control and ETBF-infected mice at week 1 and 4 post-tumor cell implantation. Data are representative of three independent experiments; p < 0.05*, p < 0.005**, p < 0.0001***, ns (nonsignificant).

We recently uncovered the oncogenic impact of ETBF in breast cancer, showing that ETBF gut or breast duct colonization enhances breast cancer growth and progression (42). Here, to examine the impact of ETBF on distant metastasis of breast cancer, we used the 4T1 breast cancer syngeneic mouse model. Using a mammary intraductal (MIND) approach, 5,000 luciferase-tagged 4T1 cells were injected into the ducts of female BALB/c mice and the tumor progression over a period of 4 weeks was monitored (**Figure 1**, **Supplementary Figure S1**). To determine the mode of metastatic dissemination, mice were sacrificed at three different time points: week 1, week 2, and week 4 post-tumor cell implantation (**Figures 1A, B**). In addition to accelerated tumor growth (volume and weight), ETBF-infected mice exhibited earlier metastatic dissemination and seeding at distant sites (**Figures 1C-F**; **Supplementary Figures S1A, B**). Marked increase in lung and liver metastatic lesions were observed in the ETBF group compared to controls (**Figure 1C**). Furthermore, we conducted *in vivo* whole-body bioluminescent imaging, *ex situ* imaging of lungs and liver, *ex situ* metastatic colony formation assay, and histological analysis to validate metastatic seeding. Whole-body bioluminescent imaging showed detectable spreading of the primary breast tumor to distant visceral organs, most likely lungs and liver in addition to bones, with the ETBF group exhibiting a higher metastatic spread (**Supplementary Figures S1A, B**). Interestingly, *ex situ* imaging of liver and lungs revealed no metastatic seeding in lungs and liver at weeks 1 and 2 post-tumor cell implantation in the control group, while seeding was evident from the luminescent signals starting at week 1 post-tumor cell implantation in ETBF-colonized mice, confirming significantly more and earlier metastatic dissemination and seeding in ETBF-infected mice (**Figure 1D**, **Supplementary Figure S1B**). Similarly, in *ex vivo* metastatic cell colony formation assay, liver and lung explants from tumor-bearing mice were digested and seeded in the presence of 6-thioguanine. While no metastatic cell colonies formed in liver digests at week 1 post-tumor cell implantation, a significantly higher number of metastatic cell colonies formed at week 2 and 4 post-tumor cell implantation in the ETBF group compared to the control group. Metastatic cell colonies formed in lung digests in the ETBF group were considerably more in number and size in comparison to controls, starting at week 1 post-tumor cell implantation, suggesting that metastatic seeding in ETBF gut colonized mice started immediately after tumor implant regardless of tumor growth (**Figure 1E**). In histological sections, although micrometastasis could not be detected at weeks 1 and 2 post-tumor cell implantation, widespread inflammation and neutrophil infiltration were evident in lungs and liver of ETBF-infected mice, while large metastases were evident at week 4 post-tumor cell implantation (**Figure 1F**, **Supplementary Figure S2**). It, therefore, suggested that the gut colonization with ETBF induced systemic inflammation in the distant organs, i.e., lungs and liver, which supported metastatic seeding in these mice. We also examined the tumors of respective groups by IHC. In line with tumor progression, Ki67 expression was notably higher in the ETBF-colonized group, as was the expression of cMyc in the nuclei of tumor cells. While β-catenin was completely membrane-bound or cytoplasmic in control tumors, it was mostly localized in nuclei in the ETBF-infected group. Notch1 expression was significantly denser in the tumors of ETBF-infected mice, as was the expression of cleaved-Notch1 in the nuclei. Vascular endothelial growth factor receptor 2 (VEGFR2), a marker of tumor vasculogenesis, also showed higher expression in ETBF-infected mice (**Supplementary Figures S3A–C**). Collectively, these results show that gut colonization with ETBF accelerates breast cancer metastasis.

### ETBF enteric infection initiates systemic inflammation and prepares the premetastatic niche

Since the pattern of metastatic dissemination was indicative of a more efficient seeding in ETBF-infected mice, we hypothesized that ETBF manipulated the premetastatic niche in the liver and lungs, supporting the establishment of secondary tumors in these organs. To test our hypothesis, non-tumor-bearing mice were infected with ETBF by oral gavage and sacrificed at day 5 post-infection, when the bacteria had stably colonized the gut (**Supplementary Figure S3D**). Serum and vital organs were analyzed to uncover the ETBF-induced changes. The serum was analyzed for 29 chemokines including proinflammatory chemokines, Th17 response chemokines, and cytokines using a multiplexed ELISA. While most of the circulating cytokines did not vary significantly between the sham control and ETBF-infected groups, some interesting patterns were apparent. Of the nine Th17 responsive chemokines, IL17A and IL17E were found to be significantly upregulated in the serum of ETBF-infected mice (**Supplementary Figure S4**). Out of the six members of the IL17 family of cytokines, IL17A, IL17B, and IL17E have been found to aid in tumor growth, angiogenesis, metastasis, and chemoresistance (48–56). Of all the cytokines, a significant upsurge was observed only in IL15 and IL27p28 (**Supplementary Figure S4**). While IL15 has been shown to be associated with better prognosis in breast cancer (57), IL27p28 has been shown to be involved in growth and metastasis of triple-negative breast cancers and has been frequently detected in breast cancer patients but undetectable in normal women (58, 59). With a significant increase in IL17A, IL17E, and IL27p28 levels, the circulatory cytokine milieu thus appears to be favorable for breast cancer progression.

**Figure 2:**
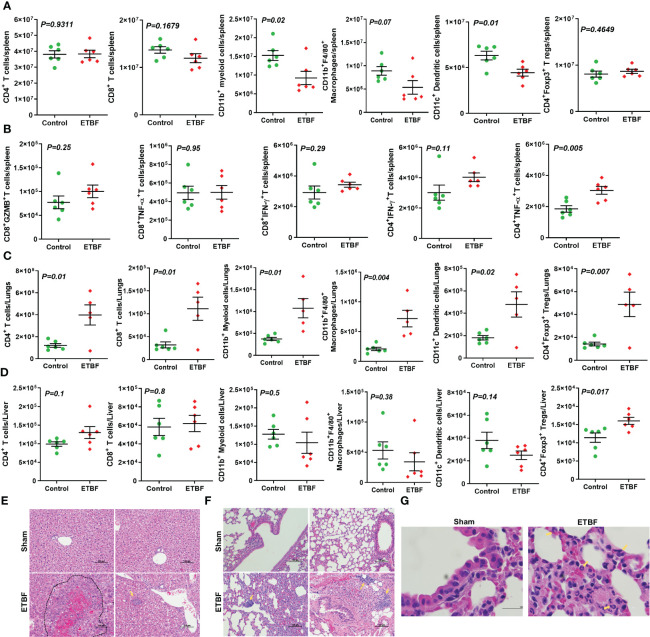
ETBF enteric infection initiates systemic inflammation and induces inflammatory cytokine production by splenocytes in non-tumor-bearing mice. **(A)** Flow cytometry data show levels of CD11b^+^ myeloid cells, CD11b^+^F4/80^+^ macrophages, CD4^+^ T cells, CD8^+^ T cells, CD11c^+^ dendritic, and CD4^+^Foxp3^+^ T regulatory cells in spleens isolated from BALB/c mice sham control or infected with ETBF for 5 days. **(B)** Graphs show flow cytometry analysis of levels of cytokines in CD8^+^ and CD4^+^ T cells in the splenocytes isolated from sham control and ETBF-infected (5 days) mice after *ex situ* stimulation with ionomycin and PMA. **(C, D)** Flow cytometry data show the levels of CD11b^+^ myeloid cells, CD11b^+^F4/80^+^ macrophages, CD4^+^ T cells, CD8^+^ T cells, CD11c^+^ dendritic, and CD4^+^Foxp3^+^ T regulatory cells in lungs and livers isolated from sham control and ETBF-infected (5 days) mice. **(E, F)** Representative H&E images of lung and liver from sham control and ETBF-infected (5 days) mice. **(G)** 100×, oil immersion images showing neutrophils and B-cell accumulation in lungs of 5-day ETBF-infected mice. Plots show data from a single cohort of mice (n = 6 mice per group). Inflammatory cells are denoted by arrows and outlined regions. Data are representative of two independent experiments analyzed separately; p < 0.05*, p < 0.005**, p < 0.0001***, ns (nonsignificant).

Next, we investigated the effect of ETBF enteric infection on systemic immune modulation. As a surrogate for systemic immune modulation, spleens were isolated from mice harboring gut ETBF infection for 5 days and analyzed by flow cytometry. In the spleen, total CD4^+^ T-cell population remained fairly unchanged along with a static population of CD4^+^Foxp3^+^ T regulatory cells and CD8^+^ T cells. However, there was a significant reduction in CD11c^+^ dendritic cells in the ETBF group, suggesting a systemic inflammatory response to ETBF infection (**Figure 2A**). There was also a statistically significant decline in the proportion of myeloid cells (CD11b^+^) and macrophages (CD11b^+^F4/80^+^) in the spleens from ETBF-infected mice. The splenocytes were then stimulated *ex situ* with phorbol 12-myristate 13-acetate (PMA) and ionomycin, and cytokine production was analyzed by flow cytometry. A significantly greater fraction of stimulated CD4^+^ T cells produced TNFα but not IFNγ in the ETBF group (**Figure 2B**). Stimulated CD8^+^ T cells trended toward increased granzyme B and IFNγ in the ETBF group (**Figure 2B**), but the difference was not statistically significant, suggesting that while the CD4^+^ T cells were activated in ETBF-infected animals compared to the controls, the status of CD8^+^ T cells remained fairly unchanged.

We then sought to find out how ETBF enteric infection shaped the immune microenvironment of liver and lungs by flow cytometry (**Figures 2C, D**). At day 5 post-ETBF infection, while lungs showed significantly higher accumulation of CD4^+^ T cells, CD4^+^Foxp3^+^ T cells, CD8^+^ T cells, CD11b^+^ myeloid cells, CD11b^+^F4/80^+^ macrophages, and CD11c^+^ dendritic cells in the ETBF group compared to the sham control group (**Figure 2C**), the liver showed higher accumulation of CD4^+^Foxp3^+^ T regulatory cells in the ETBF-infected group (**Figure 2D**). Histologic evaluation of liver and lungs from ETBF-infected mice showed interesting alterations in comparison to the sham control group. Histology of the liver showed acute-phase inflammation, microgranuloma, dead cells between neutrophils, clusters of neutrophils, hemorrhage, accumulated neutrophils, necrosis <24 h old, extramedullary hematopoiesis (EMH), and Kupffer cells in the ETBF group (**Figure 2E**). In the lungs from ETBF-infected mice, neutrophils and B cells around bronchiole and pulmonary vein were observed (**Figures 2F, G**). To closely follow the course of events, we also tested the serum from ETBF-infected, 086Mut-infected, and sham control 4T1 tumor-bearing mice at day 3 and 4 weeks post-tumor cell implantation for the levels of early-response cytokines, IL6 and TNFα. Both the cytokines showed consistent and significant upregulation from day 3 through week 4 post-tumor cell implantation in the ETBF group (**Supplementary Figure S5**). These results clearly demonstrate that ETBF gut colonization induces a systemic inflammation and prepares the premetastatic niche for the breast cancer cells.

### Tumor microenvironment remodeling in response to ETBF gut colonization

**Figure 3:**
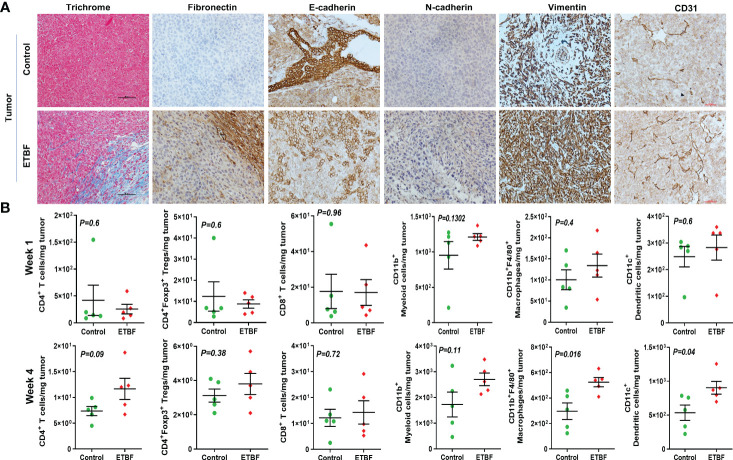
Tumor microenvironment remodeling in response to ETBF gut colonization. **(A)** Representative images of trichrome and IHC images of fibronectin, E-cadherin, N-cadherin, vimentin, and CD31 staining of intraductal 4T1 tumors developed in sham control and ETBF-infected mice. **(B)** Flow cytometry analyses of levels of CD4^+^ T cells, CD8^+^ T cells, CD11b^+^ myeloid cells, CD11b^+^F4/80^+^ macrophages, CD11c^+^ dendritic cells, and CD4^+^Foxp3^+^ T regulatory cells in intraductal 4T1 tumors developed in sham control mice or mice bearing ETBF infection for 1 week or 4 weeks. Y-axis represents the number of cells per milligram of tumor tissue. Data are representative of three independent experiments analyzed separately; p < 0.05*, p < 0.005**, p < 0.0001***, ns (nonsignificant).

**Figure 4:**
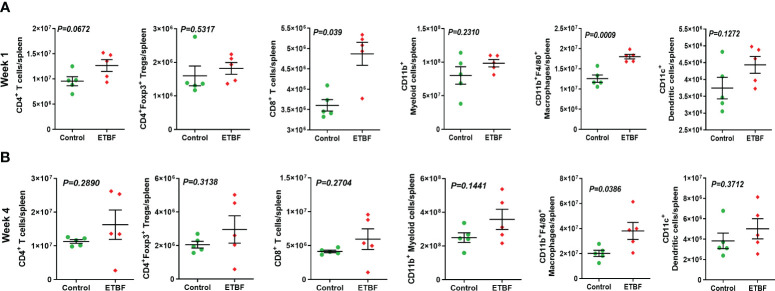
ETBF enteric infection exerts systemic immune modulation in tumor-bearing mice. **(A, B)** Flow cytometry analyses of levels of CD4^+^ T cells, CD8^+^ T cells, CD11b^+^ myeloid cells, CD11b^+^F4/80^+^ macrophages, CD11c^+^ dendritic cells, and CD4^+^Foxp3^+^ T regulatory cells in spleens of intraductal 4T1 tumor-bearing mice, sham control group or infected with ETBF for 1 week and 4 weeks. Y-axis represents the number of cells per spleen. Data are representative of three independent experiments analyzed separately; p < 0.05*, p < 0.005**, p < 0.0001***, ns (nonsignificant).

**Figure 5:**
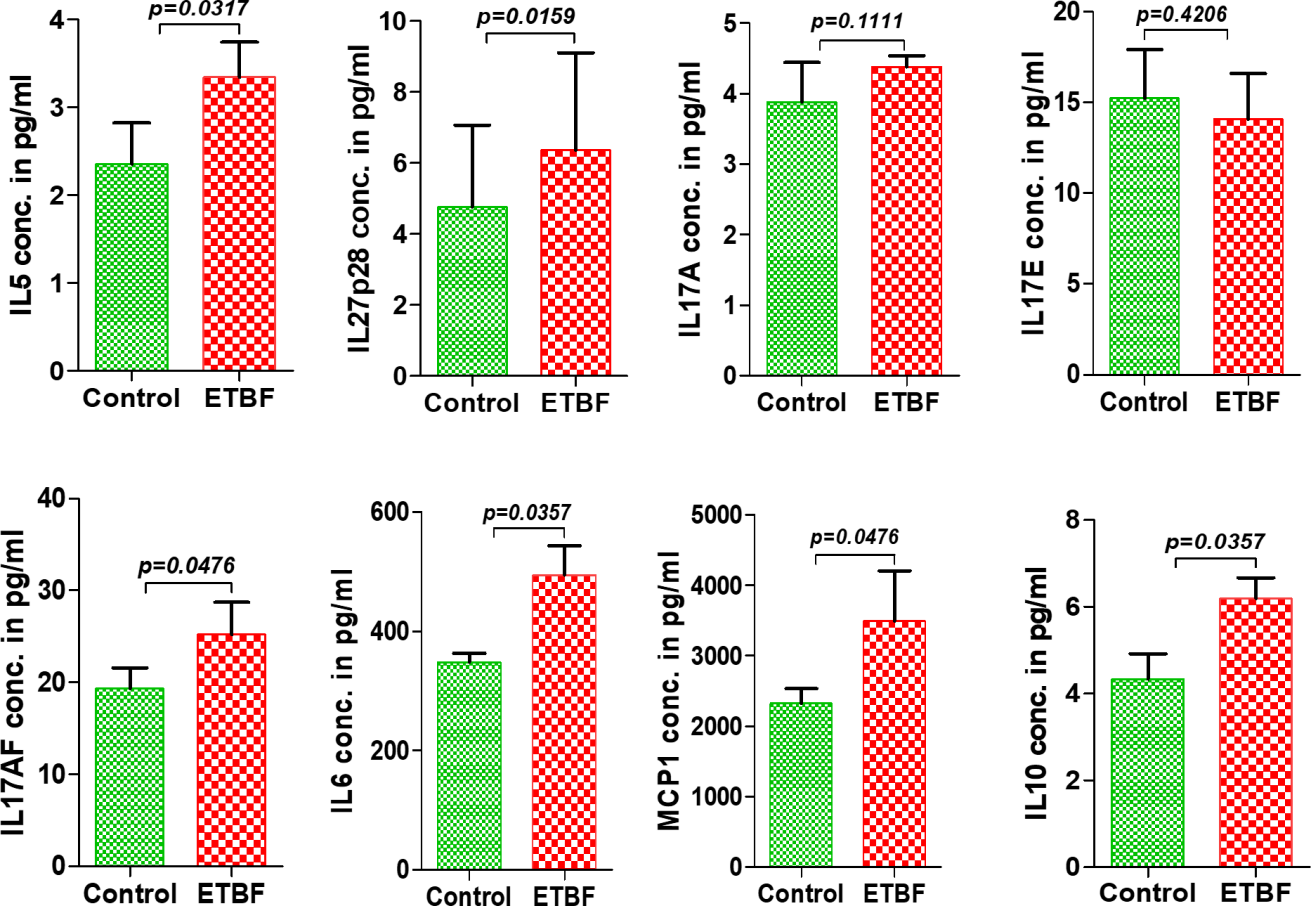
Enteric infection with ETBF increases the circulating levels of cytokines in tumor-bearing mice. Graphs show the levels of serum cytokines IL5, IL27p28, IL17A, IL17E, IL17AF, IL6, MCP1, and IL10 in intraductal 4T1 tumor-bearing mice from sham control group or ETBF-infected group post 1 and 4 weeks of tumor implant, as quantified with ELISA. Mice were colonized with 10^8^ CFUs of ETBF 3 days prior to tumor implant. Plots show data from a single cohort of mice (n = 5 mice per group). Data are representative of two independent experiments analyzed separately; p < 0.05*, p < 0.005**, p < 0.0001***, ns (nonsignificant).

Metastasis is a complex and inefficient process involving the escape of cancer cells from the primary tumors, also known as “the seed” and its establishment in distant organs, as known as “the soil.” The evolution of a tumor from benign to metastatic state involves multiple molecular mechanisms. After establishing that ETBF enteric infection causes systemic inflammation and prepares the lungs and liver as the potential metastatic sites, we asked whether it also alters the tumor microenvironment. Hence, we investigated the tumor microenvironment in both the sham control and ETBF-infected groups (**Figure 3**). Confirming the impression from H&E staining, trichrome staining showed significant stroma infiltration and fibrosis in the tumors from ETBF-infected mice (**Figure 3A**, **Supplementary Figure S6A**). Fibronectin expression was also significantly higher in the ETBF-infected group compared to tumors from the sham control group, along with higher CD31 expression showing more tumor angiogenesis (**Figure 3A**, **Supplementary Figure S6A**). While E-cadherin expression seemed to be diminished, N-cadherin was marginally higher in the ETBF-infected group. Mesenchymal marker vimentin also showed a higher expression in tumors from ETBF-infected mice in comparison to those from the sham control (**Figure 3A**, **Supplementary Figure S6A**). These results suggested that an EMT response in mammary tumors was elicited by ETBF gut infection. Next, we evaluated the immune landscape of the tumors by flow cytometry and immunohistochemistry (**Figure 3B**; **Supplementary Figures S6B, C**). Immunoblot analyses showed a higher expression of Notch as well as EMT markers in ETBF tumors compared to that in the control group (**Supplementary Figure S6D**). Tumor-infiltrating lymphocytes were evaluated by flow cytometry. While CD11b^+^ myeloid cells, CD11b^+^F4/80^+^ macrophages, and CD11c^+^ dendritic cells trended toward higher accumulation at week 1 post-tumor cell implantation in the ETBF group, CD11b^+^F4/80^+^ macrophages and CD11c^+^ dendritic cells showed significantly higher accumulation in tumors from ETBF-infected mice at 4 weeks post-tumor implantation (**Figure 3B**). Also, CD4^+^ T cells showed a notable increase (**Figure 3B**). IHC was performed to visualize the spatial organization of immune infiltrates. Macrophages and their precursors, monocytes, marked by IBA1 showed higher tumor infiltration both at week 1 and 4 post-tumor cell implantation in the ETBF group, while higher IBA1-positive cells were also observed in adjacent normal breast of ETBF-infected mice (**Supplementary Figures S7A, B**). In contrast, CD8α^+^ cell accumulation within tumor and adjacent normal breast was marginally different in ETBF-infected mice (**Supplementary Figures S7C, D**). As a surrogate for systemic immune modulation, spleens from tumor-bearing mice at 1 week and 4 weeks post-tumor cell implantation in the ETBF group were resected and analyzed by flow cytometry (**Figure 4**). Immune phenotyping showed a modulation in the proportion of CD8^+^ T cell and CD11b^+^F4/80^+^ macrophage populations at week 1 post-tumor cell implantation ETBF group. Alterations were also observed at week 4 post-tumor cell implantation ETBF group, but only CD11b^+^F4/80^+^ macrophages achieved statistical significance (**Figures 4A, B**). On interrogating the serum cytokines in these tumor-bearing mice, we observed a significant upregulation of IL5 and IL27p28 in the ETBF group (**Figure 5**), similar to the non-tumor-bearing mice, reiterating the protumorigenic role of ETBF in this breast cancer model. Unlike non-tumor-bearing mice, there was no significant alteration in the levels of IL17A and IL17E between the two groups of tumor-bearing mice, but there was a significant increase in IL17A/F, heterodimer of IL17A and IL17F, which are known to work in concert to enhance breast cancer aggressiveness (**Figure 5**). Additionally, there was a significant upregulation of prometastatic cytokines IL10, IL6, and MCP1 in the circulation in ETBF-infected mice harboring 4T1 tumors in comparison to sham control mice with 4T1 tumors (**Figure 5**). Together, these results show that ETBF gut infection leads to modulation of the tumor microenvironment.

### ETBF reshapes the metastatic niche in the liver and lungs

**Figure 6:**
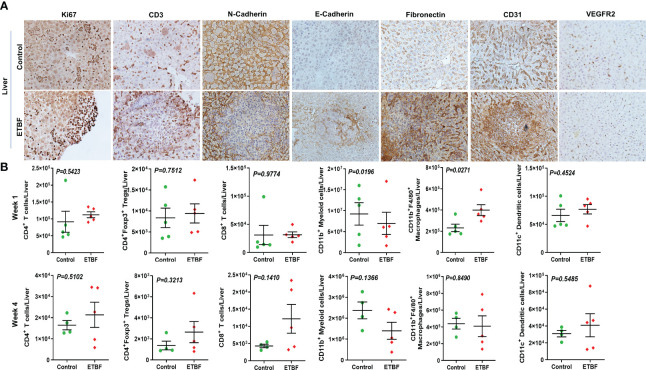
Gut colonization with ETBF reshapes the metastatic niche in the liver. **(A)** Representative images of IHC analysis of Ki67, CD3, N-cadherin, E-cadherin, fibronectin, CD31, and VEGFR2 in the livers from intraductal 4T1 tumor-bearing mice, sham control group or ETBF-infected group. **(B)** Flow cytometry analyses of levels of CD4^+^ T cells, CD8^+^ T cells, CD11b^+^ myeloid cells, CD11b^+^F4/80^+^ macrophages, CD11c^+^ dendritic cells, and CD4^+^Foxp3^+^ T regulatory cells in the livers of intraductal 4T1 tumor-bearing mice from sham control group or the group bearing enteric ETBF colonization post 1 week and 4 weeks of tumor implant. Y-axis represents the number of cells per liver. Data are representative of three independent experiments analyzed separately; p < 0.05*, p < 0.005**, p < 0.0001***, ns (nonsignificant).

**Figure 7:**
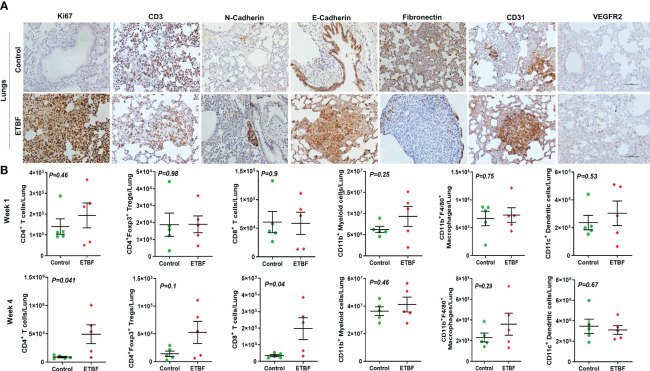
ETBF enteric infection modulates the metastatic niche in the lungs. **(A)** Representative images of IHC analysis of Ki67, CD3, N-cadherin, E-cadherin, fibronectin, CD31, and VEGFR2 in the lungs from intraductal 4T1 tumor-bearing mice, sham control or ETBF-infected. **(B)** Flow cytometry analyses of levels of CD4^+^ T cells, CD8^+^ T cells, CD11b^+^ myeloid cells, CD11b^+^F4/80^+^ macrophages, CD11c^+^ dendritic cells, and CD4^+^Foxp3^+^ T regulatory cells in the lungs of tumor-bearing mice sham-control or infected with ETBF for 1 week and 4 weeks. Y-axis represents the number of cells per liver. Data are representative of three independent experiments analyzed separately; p < 0.05*, p < 0.005**, p < 0.0001***, ns (nonsignificant).

To further investigate the mechanism of distant metastasis aggravated by ETBF enteric infection, the organs were analyzed by immunohistochemistry and flow cytometry. Metastatic lesions in the lungs and liver of ETBF-infected mice were significantly larger compared to those of the sham control group. In the liver sections, most of the metastases and small clusters of immune cells stained strongly positive for Ki67, indicating that the cells in the metastatic lesions were proliferating in the ETBF group (**Figure 6A**, **Supplementary Figure S8**). Metastatic lesions were densely surrounded by CD3^+^ immune cells in the periphery. While the primary tumors expressed little N-cadherin, the metastases expressed detectable levels of both N-cadherin and E-cadherin in the ETBF-infected mice. Interestingly, similar to primary tumors, the metastatic liver in the ETBF-infected group exhibited very high fibronectin expression. The liver of ETBF-infected 4T1 tumor-bearing mice also expressed higher levels of CD31 and VEGFR2, suggesting profuse angiogenesis (**Figure 6A**, **Supplementary Figure S8**). While IHC did not show any difference in cytotoxic CD8α^+^ T-cell accumulation in the liver, significantly higher levels of monocytes and macrophages were detected in the liver of ETBF-infected mice by IBA1-specific IHC (**Supplementary Figure S9**). By flow cytometry, statistically significant upregulation was observed only in the levels of CD11b^+^F4/80^+^ macrophages and CD11b^+^ myeloid cells at week 1 (**Figure 6B**). Scrutinizing the lung metastatic lesions, we found that most of the lungs in the ETBF gut colonized mice were filled with metastatic cancer cells and immune infiltrates, majorly neutrophils, which were far less in the sham control group (**Figure 7A**, **Supplementary Figure S10**). One hundred percent of the metastases and surrounding neutrophils stained strongly positive for Ki67 (**Figure 7A**, **Supplementary Figure S10**). In contrast, not only were the metastatic lesions smaller in the control group, they were not actively proliferating as well. While there was no difference in the density of CD3-positive cells in the lungs of the two groups (**Supplementary Figures S7**, **S10**), fibronectin level was lower in the lung metastases in the ETBF-infected group (**Figure 7A**, **Supplementary Figure S10**). Interestingly, both N-cadherin and E-cadherin levels were higher in the lung metastases in the ETBF-infected group. Mice harboring ETBF gut infection exhibited a higher expression of CD31 and VEGFR2 in the lung metastases that could support higher vasculogenesis to sustain proliferation of metastatic lesions (**Figure 7A**, **Supplementary Figure S10**). Flow cytometric analysis revealed significantly higher levels of CD8^+^ T cells and CD4^+^ T-cell infiltrates in lungs of 4-week ETBF-colonized mice, while other cell types did not vary significantly (**Figure 7B**). IHC showed a significantly higher accumulation of IBA1-positive monocytes and macrophages and marginally lower accumulation of cytotoxic CD8α^+^ T cells in the lung metastases of ETBF-infected mice in comparison to those in sham control mice (**Supplementary Figure S11**). These results unequivocally show that enteric infection with ETBF not only enhances early and efficient metastatic dissemination of cancer cells growing as primary tumors in mammary gland ducts but also causes systemic inflammation, modulates the tumor microenvironment, and primes potential metastatic niches to allow the development of metastatic lesions.

## Discussion

Recent studies have shown gut enrichment of *B. fragilis* in weight gain and obese state, and, most notably, a decrease in BMI decreases the abundance of *B. fragilis* (35–40). Obese state associates with breast cancer distant metastasis as an independent prognostic factor. A study on 18,967 early-stage breast cancer patients showed that obese women had 46% higher likelihood of developing distant metastasis within 10 years of diagnosis and 38% higher probability of death compared to their lean counterparts. While there was no effect on locoregional recurrence, response to chemotherapy as well as endocrine therapy was poorer in obese women after 10 years (60). Obesity has also been shown to drive lung and liver metastases in breast cancer patients (61). Recently, we discovered that enterotoxigenic *B. fragilis* potentiates all the hallmarks of aggressive progression in breast cancer cells, leading to increased invasion, migration, EMT, and stemness potential (42). An important part of metastatic progression is the dissemination of tumor cells from the primary source that can occur in a “linear” and/or “parallel” manner. The “linear” model supports a stepwise metastatic progression where tumor cells detach from the primary tumor, and it correlates with primary tumor size. The parallel model, however, advocates for early dissemination of tumor cells that can form metastatic lesions regardless of primary tumor size (62). We examined the impact of ETBF on the cell intrinsic and cell extrinsic factors aiding tumor cell dissemination and colonization at distant sites. This involved using a syngeneic mouse intraductal (MIND) model harboring enteric ETBF, presenting that gut colonization with ETBF is sufficient to initiate early dissemination and seeding of micrometastases to liver and lung tissue when primary tumors are barely formed. A recent study reported that another microbe, *Fusobacterium nucleatum*, specifically colonizes primary mammary tumors and promotes metastatic progression to lungs, but underlying mechanisms are unknown (20). Our results clearly support a “parallel” model where ETBF may be stimulating systemic inflammation and remodeling the premetastatic niches for efficient early metastatic dissemination.

Many microbes are known to cause persistent systemic inflammation that may lead to metastatic progression of cancer. For e.g., *Helicobacter pylori* induces inflammation through several mechanisms including the induction of proinflammatory cytokines like IL1β, IL6, IL8, and IL11 (63–65). *F. nucleatum* also evokes chronic inflammation causing alterations in the tumor microenvironment (65). Our study implicates that ETBF enteric infection initiates a systemic inflammation aiding in the establishment of premetastatic niche formation in vital organs. Interrogation of spleen as a surrogate for systemic inflammation in the state of acute infection at day 5 post-infection shows no significant change in the CD4^+^ T-cell population and the CD4^+^Foxp3^+^ T regulatory cells, while the number of CD11b^+^ myeloid cells, CD11b^+^F4/80^+^ macrophages, and CD11c^+^ dendritic cells significantly declined in the spleen. When stimulated, a significantly higher number of CD4^+^ T cells were positive for TNFα; significantly higher levels of TNFα were also detected in the circulation at every experimental time point. Breast cancer cells primarily metastasize to the lungs and liver, and examination of the landscape of these potential metastatic sites reveal interesting patterns. In the lungs, there was a significant upregulation of all classes of immune infiltrates interrogated. However, intriguingly, there was a significant increase in CD4^+^Foxp3^+^ T regulatory cells and a notable decrease in liver macrophages, myeloid cells, and dendritic cells as well, although statistically insignificant. This can be due to the fact that at 3–14 days post-ETBF infection, translocation of the bacterium from the gut to the liver has been observed at least in 25% of the cases (preliminary indicative observations, data not shown). Further experiments are needed to unequivocally decipher the mode of translocation of ETBF from the gut to various organs. Our results suggest an increased recruitment of these immune cells to the lungs and liver without an increased proliferation of splenocytes, which might aid in creating an immunosuppressive premetastatic niche in the distant organs. Confirming these observations, histological evaluation also shows an increased inflammation and EMH in the liver, while neutrophil infiltration is clearly evident in the lungs. Neutrophils actively support various processes of metastatic cascade including the formation of the premetastatic niche and are known to be induced by IL-17 levels (66). Of note, ETBF enteric infection results in a systemic IL17 response.

Investigating the microbe-induced changes in the tumor microenvironment and how that relates to the corresponding metastatic niche, we show a clearly enhanced stromal infiltration complemented with increased fibronectin and cMyc expression in the tumor sections. Reorganization of the tumor matrix components with increased tissue stiffness and stromal infiltration is known to aid in the metastatic progression of breast tumors (67). Increased fibronectin expression of breast cancer cells is known to be associated with an invasive phenotype and has also been shown to be associated with increased breast cancer mortality (68). We show that an invasive phenotype of the tumor, stimulated by enteric ETBF infection, is further supported by decreased E-cadherin and increased N-cadherin and vimentin expression, as well as nuclear accumulation of β-catenin and cleaved Notch1. The tumors are also found to be richly vascularized as evident from CD31 and VEGFR2 staining and increased proliferation indicated by Ki67 staining. The immune infiltrates play a very important role in shaping the tumor microenvironment, influence metastatic outgrowth, as well as determine therapeutic response. Slightly higher percentages of CD4^+^ T cells and myeloid cells are observed at least in the initial phase of tumor growth. While flow cytometry does not show any change in intratumoral macrophages, immunohistochemistry for IBA1, a marker for murine macrophages and precursor monocytes, shows significantly higher accumulation in the ETBF-infected tumors. No change in CD8^+^α T-cell infiltrates is observed. The tumor-associated myeloid cells, which include both Tumor associated macrophages (TAMs) and Myeloid derived suppressor cells (MDSCs), are thought to mediate breast tumor progression via immune, non-immune, and metabolic alterations, hence can potentially be targeted for improved immunotherapy outcomes.

We earlier demonstrated that ETBF, by virtue of its toxin BFT, enhances breast cancer progression, migration, metastasis, and self-renewal by inducing an EMT response in breast cancer cells via β-catenin and Notch1 pathways (42). The current study demonstrates that in addition to triggering the tumor cells, ETBF induces systemic inflammation and primes premetastatic niches in the lungs and liver, enabling successful early colonization by circulating tumor cells. ETBF induces a persistent upsurge of cytokine IL27p28, which is known to encourage dissemination of breast tumor cells and promote intratumoral myeloid cell infiltration, thereby increasing breast tumor proliferation and metastasis. IL27p28 is a pleotropic growth factor—its function ranging from proliferation of cancer cells; expression of cytokines, chemokines, growth factors, and immunomodulatory molecules on cancer cells; epithelial to immune cell-like transition of cancer cells; upregulation of oncogenes and stemness genes; and increase in tumor vascularization via crosstalk with myeloid cells (69). In breast cancer, IL30 expression in myeloid cells of tumor draining lymph nodes has been found to be an independent predictor of poor prognosis (58). Additionally, a systemic IL17 response is elicited upon ETBF enteric infection, which has been shown to stimulate myeloid cell mobilizing factors leading to increased myeloid cell recruitment into the tumors that further increases tumor progression via increased angiogenesis and suppression of antitumor immune response. We noted that gut colonization with ETBF is heterogenous, and a remaining question is whether there is a direct correlation between the extent of gut colonization with ETBF and site-specific immune changes. Another question that requires further examination is the contribution of gut versus breast ETBF infection in mediating the site-specific changes in the immune microenvironment and metastatic progression. It has not been feasible to tease out the effect of gut ETBF infection exclusively largely because we observed the presence of ETBF in mammary ducts of mice harboring gut ETBF infection, although the mode of transportation is unclear. Directly infecting the mammary ductal system via injecting ETBF intraductally can potentially help isolate the impact of ETBF harbored in the mammary gland and show whether breast ETBF is crucial for creating metastatic niches in the lungs and liver and support aggressive metastatic progression.

## Conclusions

The intricate interactions between breast cancer, procarcinogenic bacterium ETBF, and the immune system are not yet fully understood. Our data suggest that ETBF gut colonization elicits a systemic immune response leading to an upsurge of IL27p28 and IL17 cytokines, which, in addition to shaping the lung and liver premetastatic niches, interacts with the myeloid rich tumor microenvironment enhancing the metastatic progression of breast cancer. Our study agrees with the “seed and soil hypothesis” as the ETBF–BFT axis directly modulates cancer cells (seed) as well as metastatic niches (soil). It also supports the “parallel model” of metastasis advocating for an early dissemination of tumor cells that form metastatic lesions regardless of the primary tumor size.

## Data availability statement

The original contributions presented in the study are included in the article/**Supplementary Material**. Further inquiries can be directed to the corresponding authors.

## Ethics statement

The animal study was reviewed and approved by Johns Hopkins ACUC.

## Author contributions

Conceptualization: SP, DS; Methodology: SP, SS, HG, SW, GW, BL, KG, DS; Investigation: SP, SS, HG, SW, GW, BL, KG, DS; Formal analysis: SP, SS, HG, SW, GW, BL, KG, DS; Resources: BL, CS, DS; Data curation: SP, SS, HG, SW, GW, BL, KG, DS; Writing-original draft preparation: SP, DS; Writing-review and editing: SP, DS; Visualization: SP, DS; Supervision and project administration: SP, BL, CS, DS. All authors contributed to the article and approved the submitted version.

## Supplementary material

Supplementary Figure 1 Enteric ETBF infection enhances breast cancer aggressiveness in syngeneic mice model. **(A)** Bioluminescence images of sham-control and ETBF infected mice bearing intraductal 4T1 tumor for week 1, 2, 3 and 4. **(B)** Plots showing metastatic load in lungs and liver of 4T1 tumor bearing mice with or without enteric ETBF infection at week 1, week 2 and week 4 by bioluminescent imaging.

Supplementary Figure 2 ETBF enhances Ly6G staining. Representative IHC images for Ly6G specific staining in tumor, lungs and liver of ETBF infected and Control mice and corresponding quantification.

Supplementary Figure 3 ETBF gut colonization increases the levels of oncogenic proteins in tumors. **(A)** Representative IHC images of Ki67, β catenin, cMyc, Notch1, cleaved notch1 and VEGFR2 staining of intraductal 4T1 tumors from sham-control and ETBF-infected mice, **(B)** corresponding quantification and **(C)** and annotated images using Aperio ImageScope. **(D)** CFU counts from stools of mice infected with ETBF are presented here as a bar graph.

Supplementary Figure 4 ETBF enteric infection induces systemic inflammation. Graphs show the levels of serum cytokines in intraductal 4T1 non-tumor bearing mice (N=5), sham-control or infected with ETBF at day 5, as quantified with multiplexed ELISA. Out of 29 cytokines queried, 4 remained undetected.

Supplementary Figure 5 Mice harboring gut colonization of ETBF exhibit induction of acute phase inflammation in comparison to mice infected with 086Mutant *B. fragilis*. Graphs show the levels of serum cytokines IL6 and TNFα in intraductal 4T1 tumor bearing mice (N=5); sham-control, infected with ETBF or infected with 086Mutant *B. fragilis* at day 3 and week 4 post-tumor cell implantation time points. Data is representative of 2 independent experiments analyzed separately; *p<0.05*, p<0.005**, p<0.0001***, ns (non-significant)*

Supplementary Figure 6 ETBF enteric infection shapes the breast TME. **(A)** Aperio images scope annotated images and corresponding quantification of , **(B)** Representative images and quantification of CD45 specific IHC in sections of intraductal 4T1 tumors developed in sham-control and ETBF-infected mice. **(C)** Graphs showing number of Tumor infiltrating lymphocytes per mg tumor and number of viable CD45^+^ TILS/mg of tumor isolated in respective groups of representative experiments presented in the study. **(D)** Western blot analysis with corresponding quantification showing up-regulation of Notch-1 signaling and mesenchymal markers in tumors from ETBF infected mice compared to sham-treated mice.

Supplementary Figure 7 ETBF enteric infection modulates the expression of monocyte and macrophage specific antigen IBA1 and cytotoxic T cell specific antigen CD8α. **(A, B)** Representative IHC images and corresponding quantification of IBA1 in tumor tissues, lymph nodes and normal mammary gland tissues in sham-control and ETBF-infected mice at week 1 and week 4 post-tumor cell implantation time points. **(C, D)** Representative IHC images and corresponding quantification of CD8α in tumor tissues and normal mammary gland tissues in sham-control and ETBF-infected mice at week 1 and week 4 post-tumor cell implantation time points.

Supplementary Figure 8 Enteric ETBF infection modulates the spatial organization of immune cells in the liver. **(A, B)** Aperio images scope annotated images and corresponding quantification of **Figure 6A**.

Supplementary Figure 9 Enteric ETBF infection modulates the spatial organization of immune cells in the liver. **(A)** Representative IHC images, **(B)** Aperio images scope annotated images and **(C)** corresponding quantification showing the expression of hematopoietic lineage marker CD45, monocyte and macrophage specific antigen IBA1 and T cell specific antigen CD8α in livers of sham-control and ETBF-infected mice.

Supplementary Figure 10 Enteric ETBF infection modulates the spatial organization of immune cells in the lungs. **(A)** Aperio images scope annotated images and **(B)** corresponding quantification of **Figure 7A**.

Supplementary Figure 11 Enteric ETBF infection modulates the spatial organization of immune cells in the lungs. **(A)** Representative IHC images, **(B)** Aperio images scope annotated images and **(C)** corresponding quantification showing the expression of hematopoietic lineage marker CD45, monocyte and macrophage specific antigen IBA1 and T cell specific antigen CD8α in the lungs of sham-control and ETBF-infected mice.
