# Supplementary figures and images for: Gut colonization with an obesity-associated enteropathogenic microbe modulates the premetastatic niches to promote breast cancer lung and liver metastasis
Source: Front Immunol. 2023 Jul 12;14:1194931. doi: 10.3389/fimmu.2023.1194931 (PMC10369066; doi:10.3389/fimmu.2023.1194931)

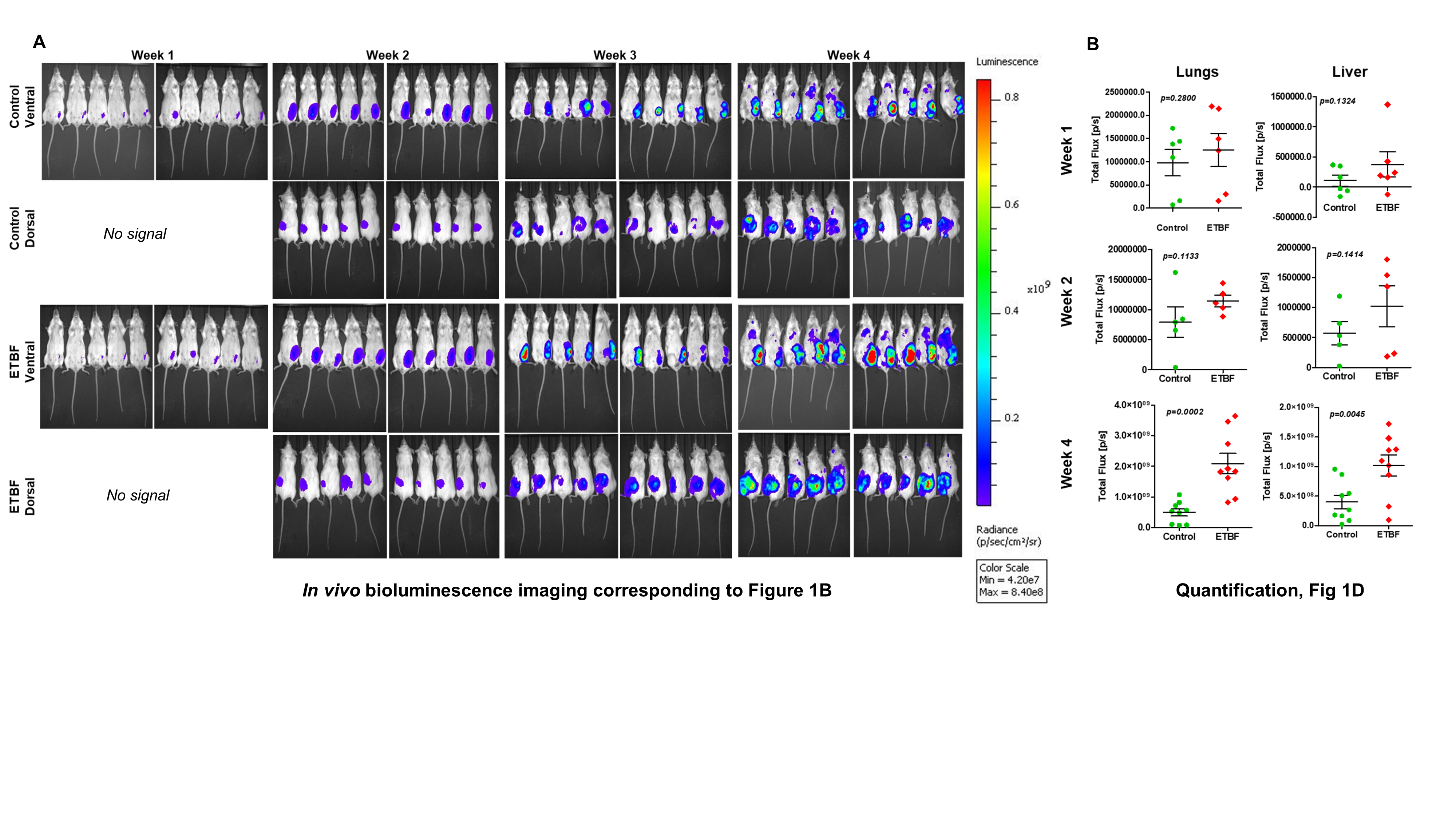

Supplement: Supplementary Figure 1 — Enteric ETBF infection enhances breast cancer aggressiveness in syngeneic mice model. (A) Bioluminescence images of sham-control and ETBF infected mice bearing intraductal 4T1 tumor for week 1, 2, 3 and 4. (B) Plots showing metastatic load in lungs and liver of 4T1 tumor bearing mice with or without enteric ETBF infection at week 1, week 2 and week 4 by bioluminescent imaging. [file Image_1.tif]

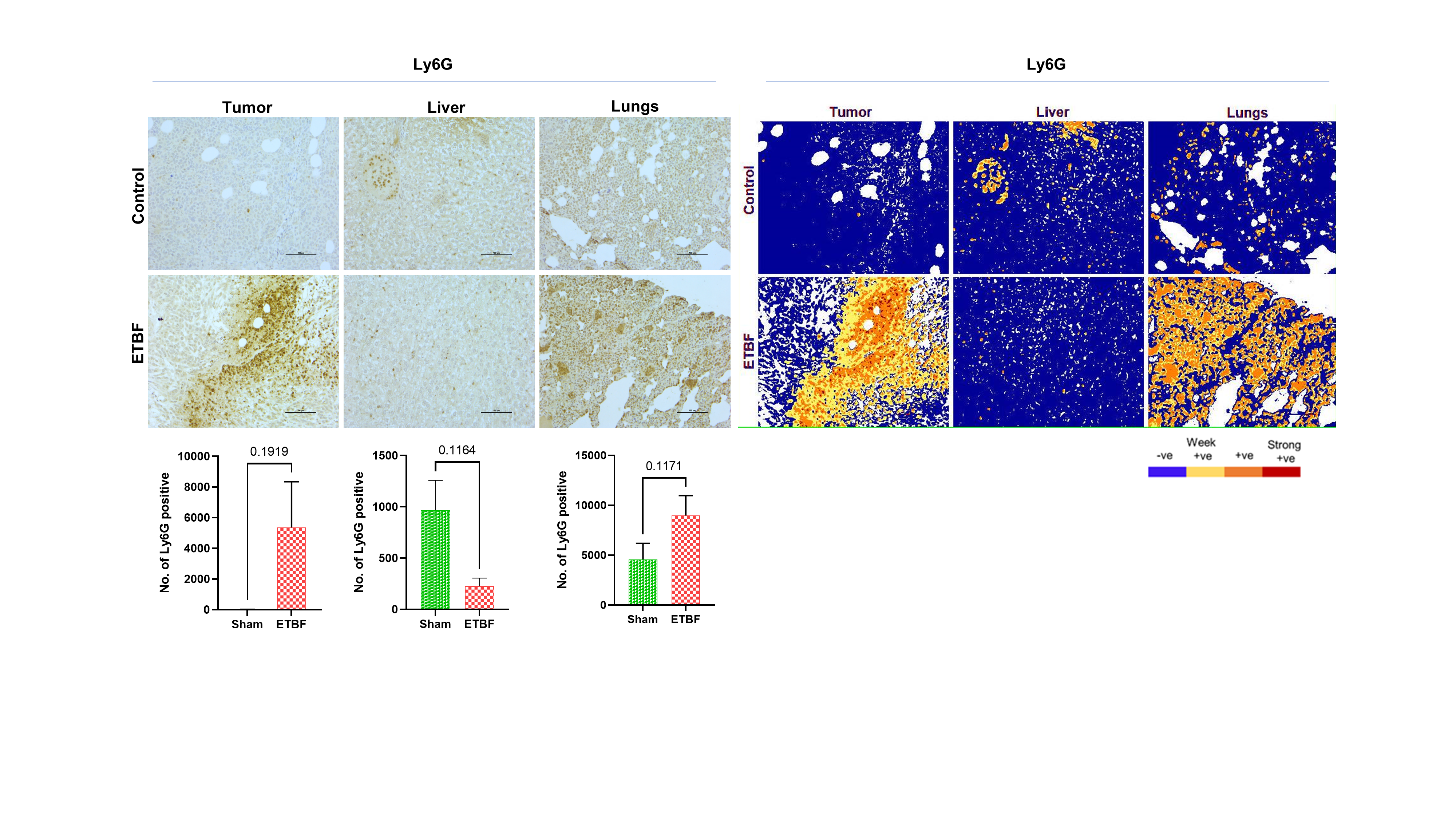

Supplement: Supplementary Figure 2 — ETBF enhances Ly6G staining. Representative IHC images for Ly6G specific staining in tumor, lungs and liver of ETBF infected and Control mice and corresponding quantification. [file Image_2.tif]

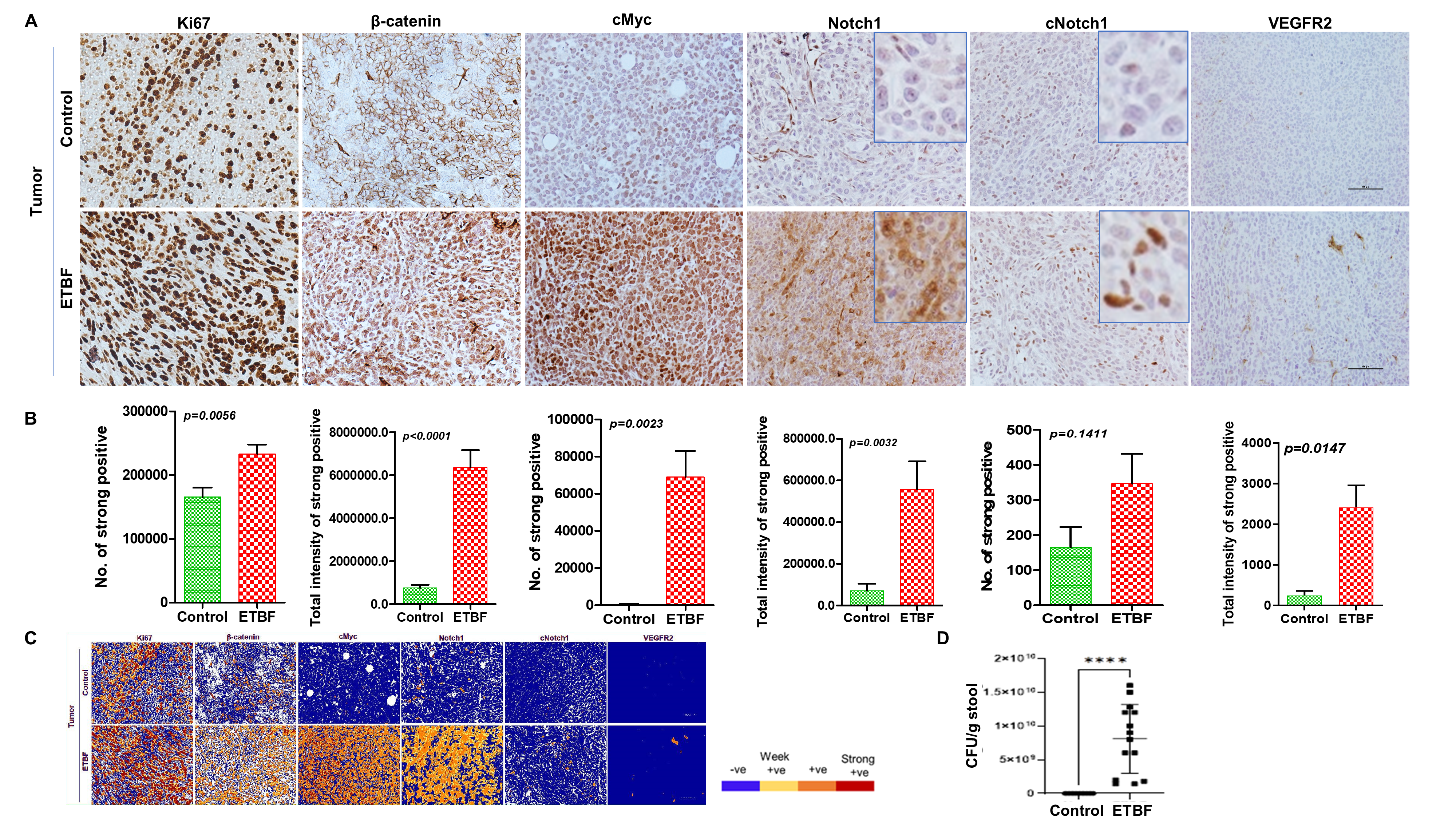

Supplement: Supplementary Figure 3 — ETBF gut colonization increases the levels of oncogenic proteins in tumors. (A) Representative IHC images of Ki67, β catenin, cMyc, Notch1, cleaved notch1 and VEGFR2 staining of intraductal 4T1 tumors from sham-control and ETBF-infected mice, (B) corresponding quantification and (C) and annotated images using Aperio ImageScope. (D) CFU counts from stools of mice infected with ETBF are presented here as a bar graph. [file Image_3.tif]

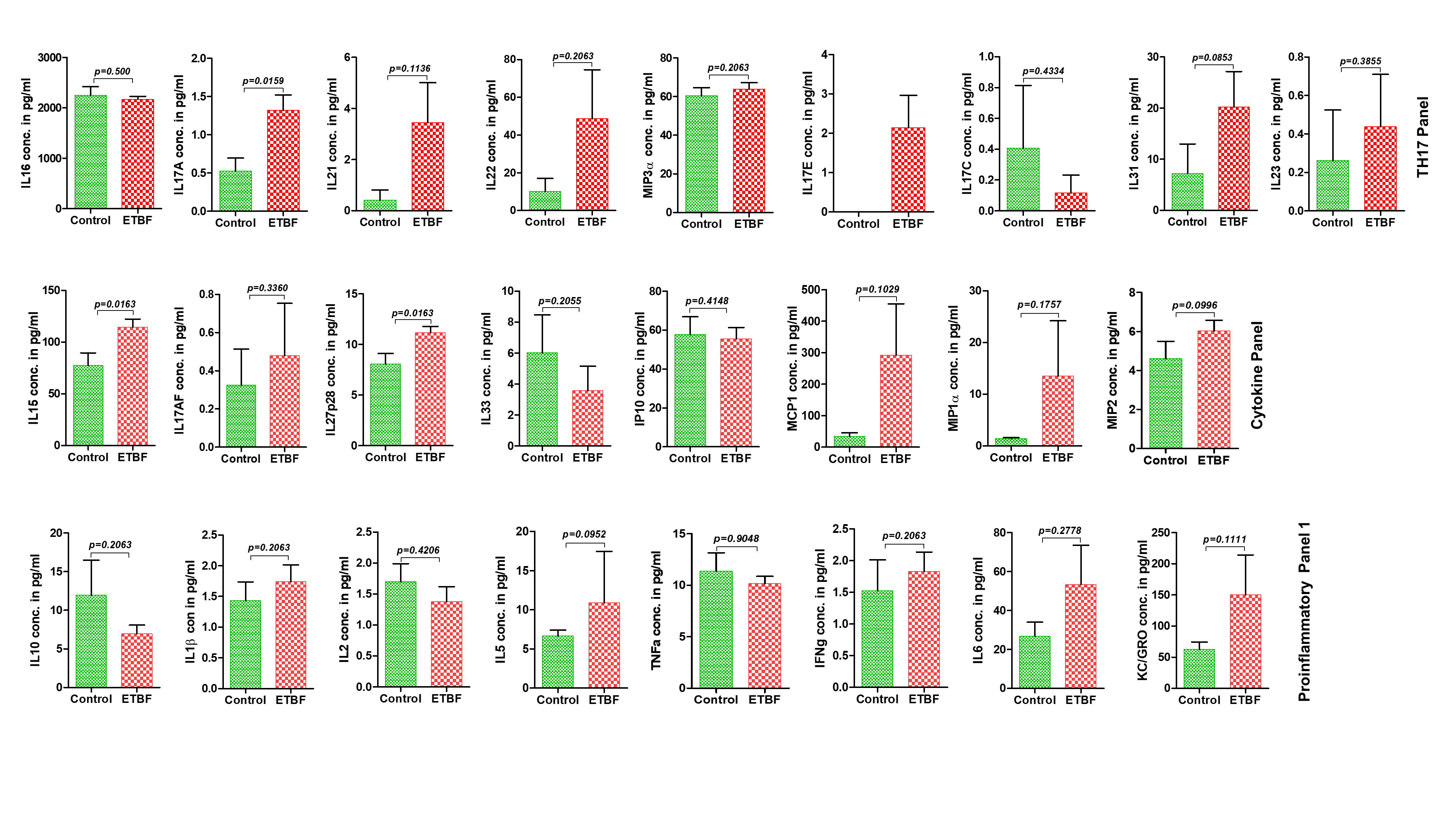

Supplement: Supplementary Figure 4 — ETBF enteric infection induces systemic inflammation. Graphs show the levels of serum cytokines in intraductal 4T1 non-tumor bearing mice (N=5), sham-control or infected with ETBF at day 5, as quantified with multiplexed ELISA. Out of 29 cytokines queried, 4 remained undetected. [file Image_4.tif]

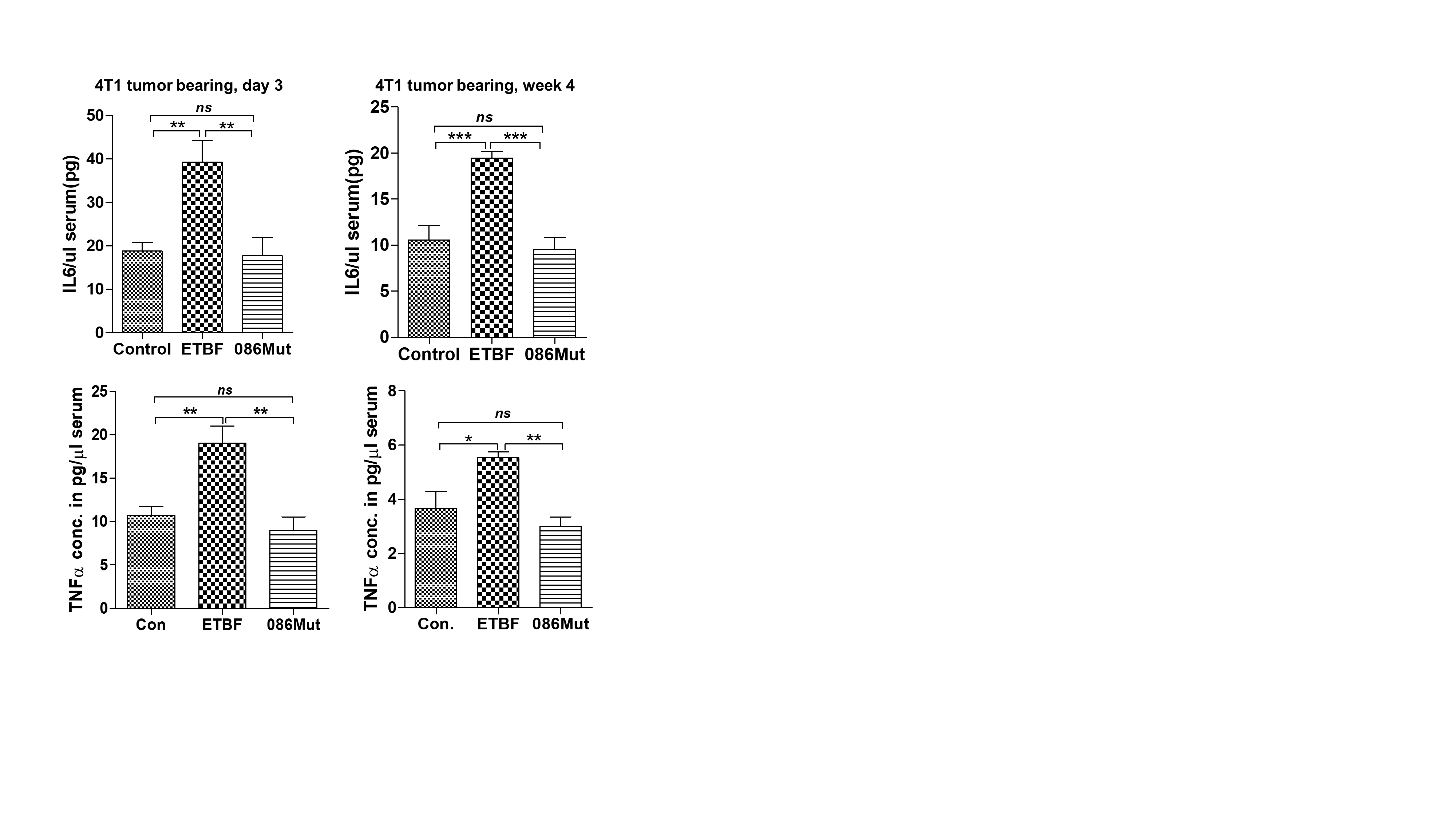

Supplement: Supplementary Figure 5 — Mice harboring gut colonization of ETBF exhibit induction of acute phase inflammation in comparison to mice infected with 086Mutant B. fragilis. Graphs show the levels of serum cytokines IL6 and TNFα in intraductal 4T1 tumor bearing mice (N=5); sham-control, infected with ETBF or infected with 086Mutant B. fragilis at day 3 and week 4 post-tumor cell implantation time points. Data is representative of 2 independent experiments analyzed separately; p<0.05*, p<0.005**, p<0.0001***, ns (non-significant) [file Image_5.tif]

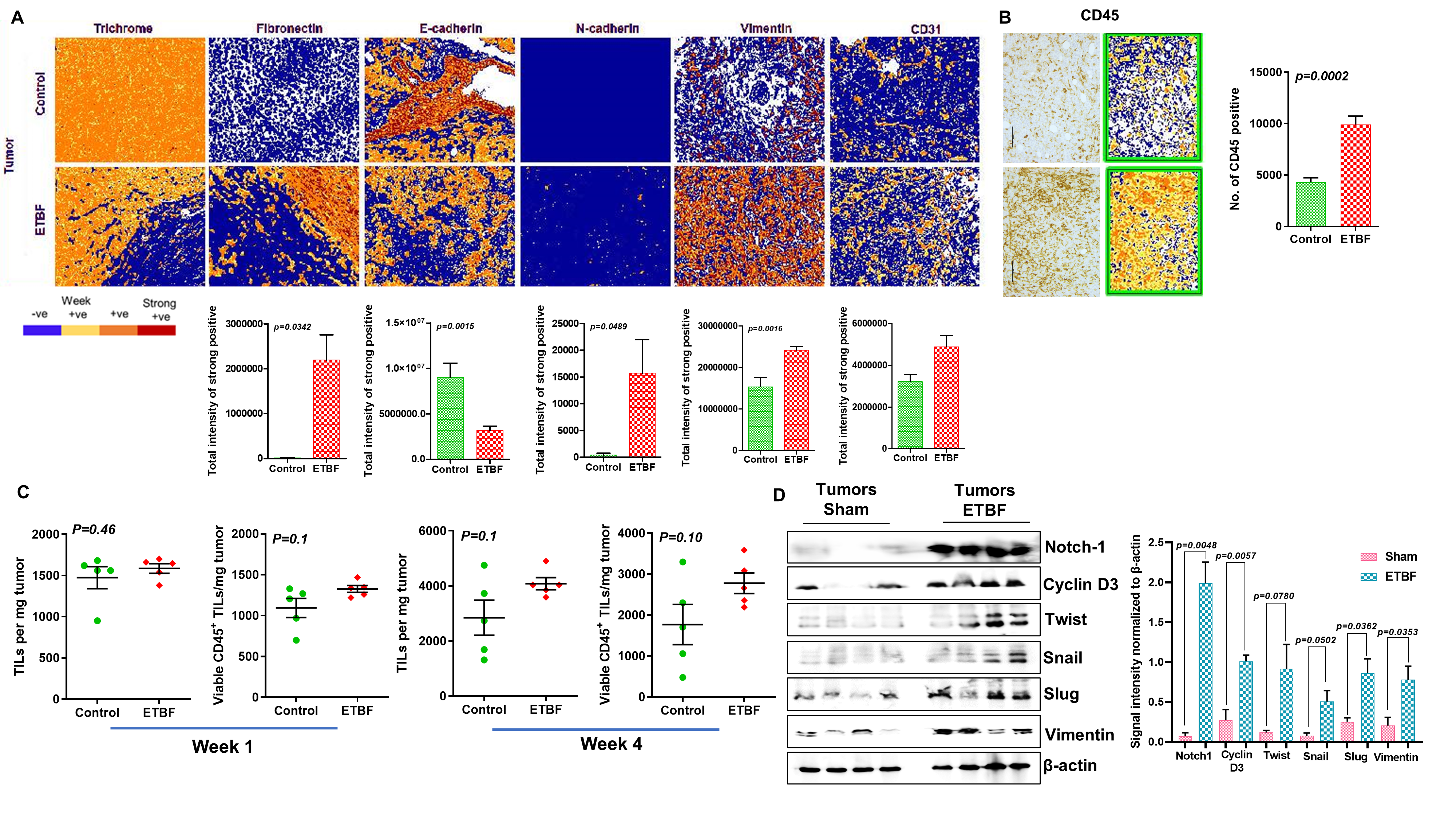

Supplement: Supplementary Figure 6 — ETBF enteric infection shapes the breast TME. (A) Aperio images scope annotated images and corresponding quantification of , (B) Representative images and quantification of CD45 specific IHC in sections of intraductal 4T1 tumors developed in sham-control and ETBF-infected mice. (C) Graphs showing number of Tumor infiltrating lymphocytes per mg tumor and number of viable CD45+ TILS/mg of tumor isolated in respective groups of representative experiments presented in the study. (D) Western blot analysis with corresponding quantification showing up-regulation of Notch-1 signaling and mesenchymal markers in tumors from ETBF infected mice compared to sham-treated mice. [file Image_6.tif]

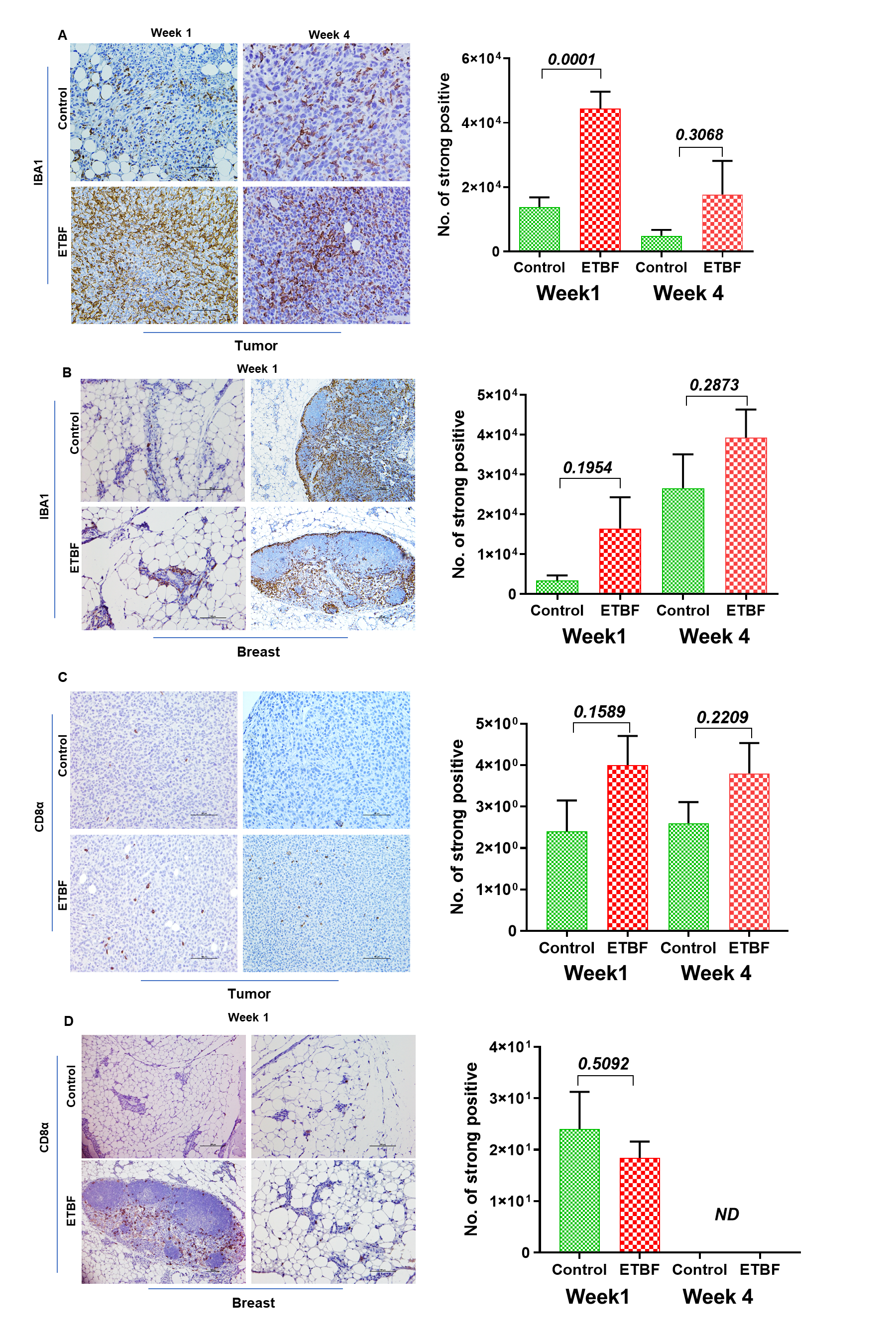

Supplement: Supplementary Figure 7 — ETBF enteric infection modulates the expression of monocyte and macrophage specific antigen IBA1 and cytotoxic T cell specific antigen CD8α. (A, B) Representative IHC images and corresponding quantification of IBA1 in tumor tissues, lymph nodes and normal mammary gland tissues in sham-control and ETBF-infected mice at week 1 and week 4 post-tumor cell implantation time points. (C, D) Representative IHC images and corresponding quantification of CD8α in tumor tissues and normal mammary gland tissues in sham-control and ETBF-infected mice at week 1 and week 4 post-tumor cell implantation time points. [file Image_7.tif]

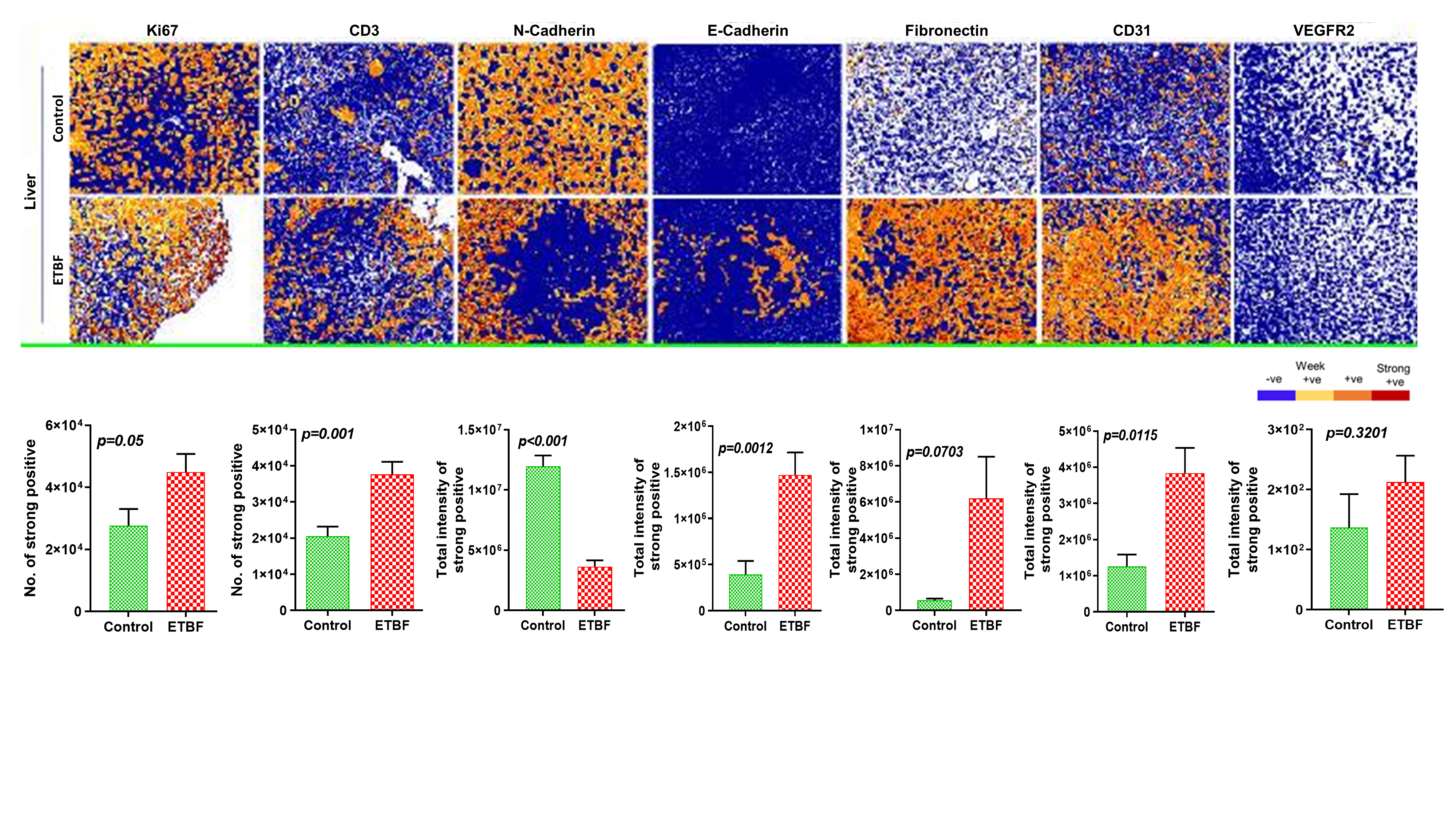

Supplement: Supplementary Figure 8 — Enteric ETBF infection modulates the spatial organization of immune cells in the liver. (A, B) Aperio images scope annotated images and corresponding quantification of Figure 6A . [file Image_8.tif]

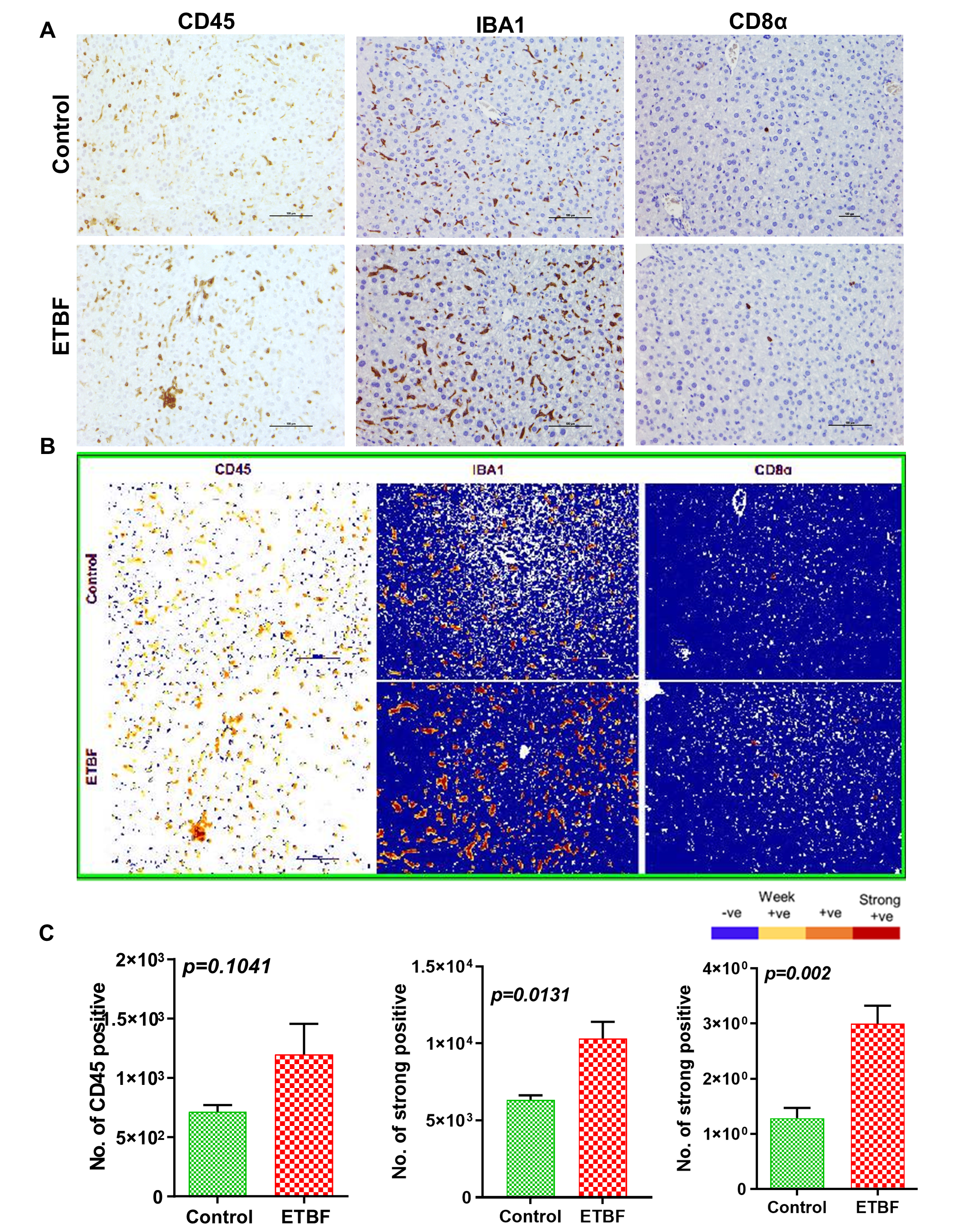

Supplement: Supplementary Figure 9 — Enteric ETBF infection modulates the spatial organization of immune cells in the liver. (A) Representative IHC images, (B) Aperio images scope annotated images and (C) corresponding quantification showing the expression of hematopoietic lineage marker CD45, monocyte and macrophage specific antigen IBA1 and T cell specific antigen CD8α in livers of sham-control and ETBF-infected mice. [file Image_9.tif]

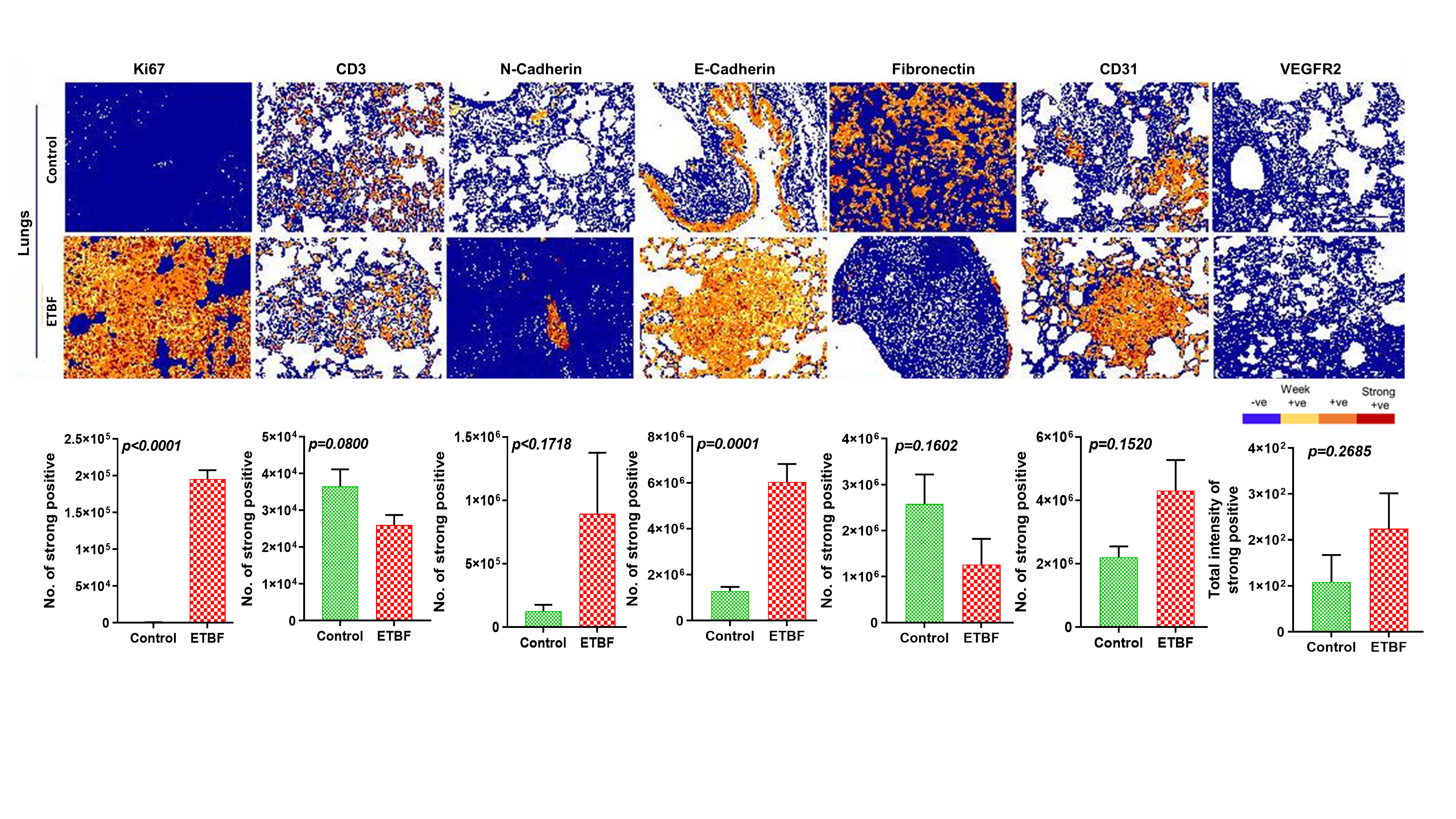

Supplement: Supplementary Figure 10 — Enteric ETBF infection modulates the spatial organization of immune cells in the lungs. (A) Aperio images scope annotated images and (B) corresponding quantification of Figure 7A . [file Image_10.tif]

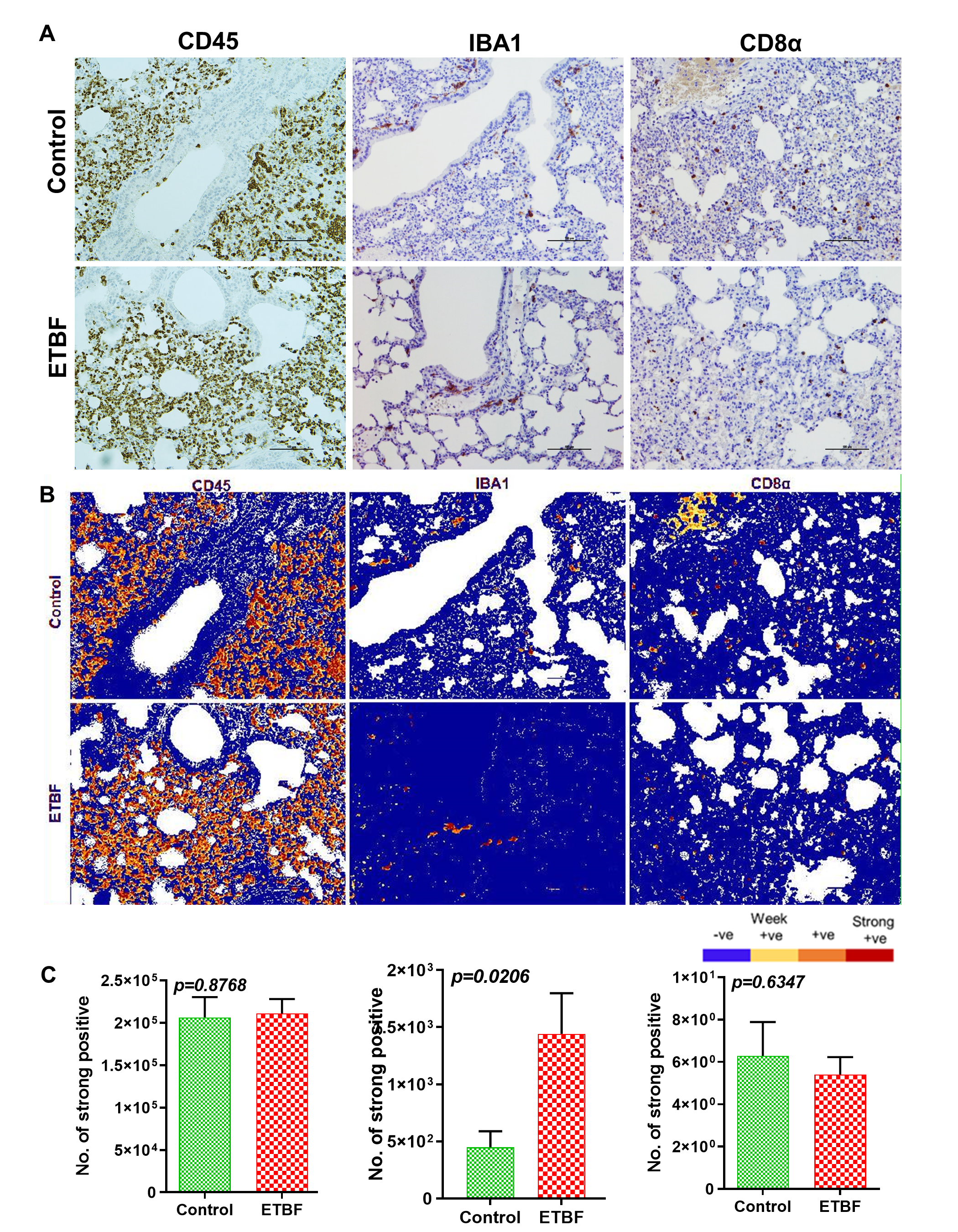

Supplement: Supplementary Figure 11 — Enteric ETBF infection modulates the spatial organization of immune cells in the lungs. (A) Representative IHC images, (B) Aperio images scope annotated images and (C) corresponding quantification showing the expression of hematopoietic lineage marker CD45, monocyte and macrophage specific antigen IBA1 and T cell specific antigen CD8α in the lungs of sham-control and ETBF-infected mice. [file Image_11.tif]
